# Efficient and markerless gene integration with SlugCas9-HF in *Kluyveromyces marxianus*

**DOI:** 10.1038/s42003-024-06487-w

**Published:** 2024-07-02

**Authors:** Huanyu Zhou, Tian Tian, Jingtong Liu, Hong Lu, Yao Yu, Yongming Wang

**Affiliations:** 1 Center for Medical Research and Innovation, Shanghai Pudong Hospital, Fudan University Pudong Medical Center, Fudan University, Shanghai, 201399 China; 2 State Key Laboratory of Genetic Engineering, School of Life Sciences, Shanghai Engineering Research Center of Industrial Microorganisms, Fudan University, Shanghai, 200438 China

**Keywords:** Agriculture, CRISPR-Cas9 genome editing

## Abstract

The nonconventional yeast *Kluyveromyces marxianus* has potential for industrial production, but the lack of advanced synthetic biology tools for precise engineering hinders its rapid development. Here, we introduce a CRISPR-Cas9-mediated multilocus integration method for assembling multiple exogenous genes. Using SlugCas9-HF, a high-fidelity Cas9 nuclease, we enhance gene editing precision. Specific genomic loci predisposed to efficient integration and expression of heterologous genes are identified and combined with a set of paired CRISPR-Cas9 expression plasmids and donor plasmids to establish a CRISPR-based biosynthesis toolkit. This toolkit enables genome integration of large gene modules over 12 kb and achieves simultaneous quadruple-locus integration in a single step with 20% efficiency. As a proof-of-concept, we apply the toolkit to screen for gene combinations that promote heme production, revealing the importance of *HEM4Km* and *HEM12Sc*. This CRISPR-based toolkit simplifies the reconstruction of complex pathways in *K. marxianus*, broadening its application in synthetic biology.

## Abstract (web summary)

Fungi has been widely used as protein and drug production factories, in which efficient heterologous gene expression is fundamental. This paper provides a genome editing tool kit for heterologous gene expression in *Kluyveromyces marxianus*.

## Introduction

Yeasts are a diverse group of eukaryotic fungi that have long been employed as cell factories. The number of yeast species with specific advantages continues to grow. Among them, the conventional yeast S*accharomyces cerevisiae* stands out as the most widely utilized eukaryotic cell factory due to its availability, genetic tractability, well-established physiology, and convenient genetic manipulation^1^. Nevertheless, nonconventional yeast species with favorable characteristics for industrial bioprocesses are gaining significance. One such yeast, *Kluyveromyces marxianus*, is particularly intriguing due to its capacity to utilize a wide range of carbon sources (including lactose, glucose, xylose, inulin, arabinose, and galactose)^2^, its thermotolerance (>40 °C)^3^, secretion of lytic enzymes (such as β-galactosidase, pectinase, and inulinase)^4^, its rapid growth rate compared to other eukaryotes^1^, and its ability to produce fuel ethanol through fermentation^5^. These desirable traits position *K. marxianus* as a versatile host for applications in the food^6^, feed^7^, and pharmaceutical industries^8^.

Currently, CRISPR-Cas9 technology is being used to engineer *K. marxianus* for heterologous gene expression^9^. CRISPR-Cas9 is a two-component system consisting of a Cas9 nuclease and a single guide RNA (sgRNA)^10–13^. Cas9 and sgRNA form a ribonucleoprotein complex that binds to and cuts the target DNA complementary to the 5’ end of the sgRNA, generating a double-strand break (DSB)^10^. To repair the DSBs, cells either utilize non-homologous end-joining (NHEJ), which results in nonspecific small insertions and deletions (indels) useful for generating loss-of-function mutations or utilize homology-directed repair (HDR) using an introduced DNA repair template, such as a double-stranded DNA donor plasmid or a single-stranded oligo DNA nucleotide (ssODN), leading to the precise knock-in of mutations or heterologous genes^14,15^. Juergenes et al. achieved over 80% disruption of *ADE2* and 24% HDR-based repair in *K. marxianus* haploid and diploid strains using CRISPR-Cas9 technology^16^. Since NHEJ likely plays a major role in *K. marxianus*, Nambu-Nishida et al. disrupted the cell type-specific regulator (Nej1) and DNA ligase 4 (Dnl4) to repress NHEJ, resulting in 100% HDR-mediated genome editing at the *URA3* site^17^.

Several genomic loci for efficient heterologous gene integration and expression have been previously identified in *K. marxianus*^18,19^. However, these loci are located within coding genes, disrupting gene function upon heterologous gene integration. In this study, we utilized SlugCas9-HF, which exhibited high editing efficiency and specificity^20^, to screen a panel of genomic loci located within intergenic regions. Through this approach, we identified six loci suitable for efficient heterologous gene integration and expression in *K. marxianus*. Furthermore, we demonstrated that these loci allowed for simultaneous multigene integration, thereby promoting heme biosynthesis. The CRISPR-Cas9 expression plasmids and donor plasmids developed in this study provide a convenient platform for simple, efficient, and rapid engineering of *K. marxianus* for biosynthesis purposes.

## Results

### Genome editing with SlugCas9-HF in *K. marxianus*

**Fig. 1:**
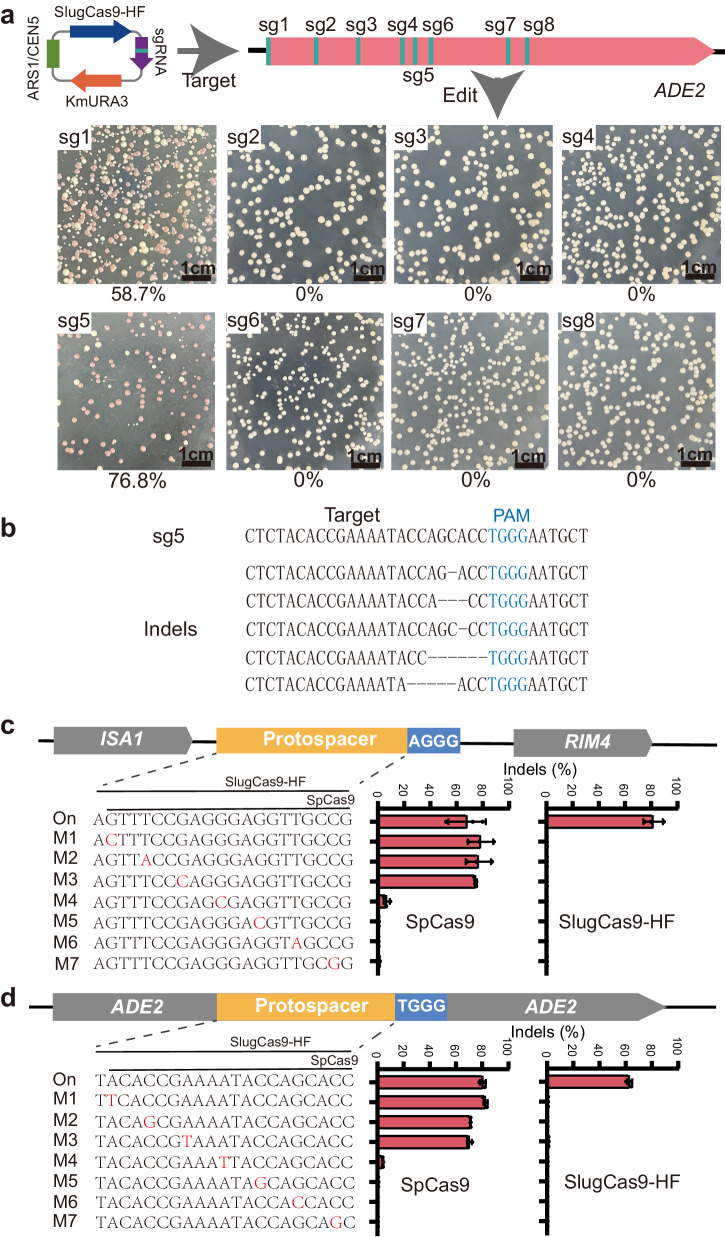
SlugCas9-HF enables efficient and precise genome editing in *K. marxianus.* **a** SlugCas9-HF-mediated disruption of *ADE2*. The top panel illustrates the editing plasmids and target sites in the *ADE2* gene. Yeast colonies after genome editing are depicted below, with red colonies indicating successful disruption of the *ADE2* gene. The percentage of red colonies is indicated below the images. **b** Deep sequencing results demonstrating SlugCas9-HF-induced indels at the target sites. **c**, **d** Specificity analysis of SpCas9 and SlugCas9-HF. The top panel shows the schematic of the target site. A panel of sgRNAs with single nucleotide mutations is presented below. Each sgRNA’s activity for SpCas9 and SlugCas9-HF was assessed based on indel rates (*n* = 3). Error bars represent the mean ± SD of biological triplicates.

First, we assessed the functionality of SlugCas9-HF in *K. marxianus*. The hybrid RNA polymerase III (Pol III) promoter RPR1-tRNA^gly^, known for facilitating high gene expression, was employed to express sgRNA (LHZ1493 plasmid)^21^. The sgRNA and SlugCas9-HF were then cloned into a centromere vector in *K. marxianus*, resulting in an editing plasmid with a *URA3* marker. We designed eight sgRNAs (sg1-sg8) targeting the adenine biosynthesis pathway gene *ade2*. Yeast cells with a mutant *ade2* allele (*∆ade2*) appear to have a red phenotype on the YPD medium owing to the accumulation of a red intermediate compound in vacuoles^22^. After the transformation of editing plasmids, yeast cells were allowed to grow on a YPD solid medium to form colonies over two days. Interestingly, colonies with an obvious accumulation of a red pigment were observed in two plates (sg1 and sg5), indicating successful genome editing (Fig. 1a). The sg1 and sg5 sgRNAs achieved 58.7% and 76.8% red colonies, respectively. To confirm genome editing, we randomly picked up 100 red colonies from the sg5 plate, pooled them together, and extracted genomic DNA for targeted deep sequencing. Sequencing results showed that indels occurred (Fig. 1b), demonstrating the functionality of SlugCas9-HF in *K. marxianus*.

Furthermore, we examined the impact of Cas9 expression on the growth of *K. marxianus*. The growth curve of yeast cells harboring vector plasmids, with or without SlugCas9-HF, exhibited similar growth rates, suggesting that Cas9 expression does not negatively affect the growth of *K. marxianus* (Supplementary Fig. 1).

Off-target effects are always a major concern as they can lead to unintended mutations in the genome. While SlugCas9-HF has demonstrated high specificity in mammalian cells^20^, its specificity in yeast remains unknown. To explore the specificity of SlugCas9-HF in *K. marxianus*, we selected a genomic site between *ISA1* and *RIM4* genes and a genomic site on the *ADE2* gene. Two panels of sgRNAs with single nucleotide mutations along the protospacer were designed (Fig. 1c, d). SpCas9 was used for comparison. Two days after yeast transformation, we extracted genomic DNA for targeted deep sequencing. The sequencing results showed that both Cas9 nucleases induced robust and comparable editing efficiency. SpCas9 tolerated single mismatches at the PAM-distal region, while SlugCas9-HF did not tolerate single mismatches along the protospacer. These findings demonstrate that SlugCas9-HF exhibits high specificity in *K. marxianus*.

### The screen of potential gene integration sites

**Fig. 2:**
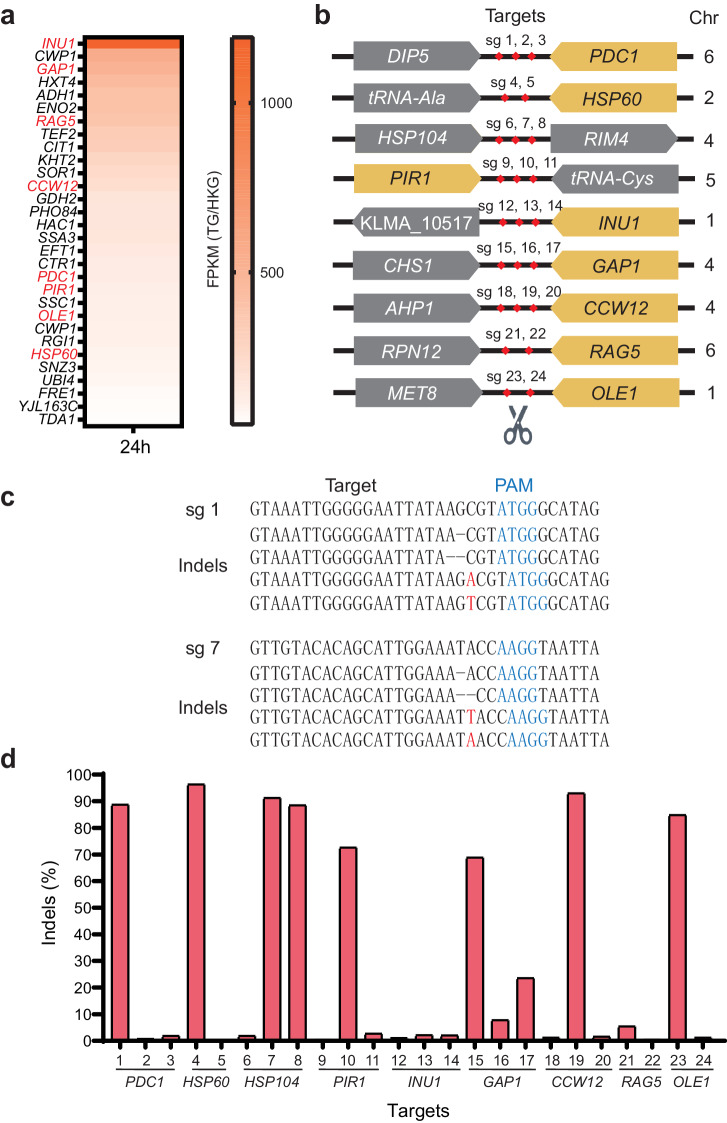
The screen of gene integration sites. **a** Identification of the top 30 highly expressed genes in *K. marxianus*. Genes were identified from a previously reported RNA-seq dataset^23^. The bar represents FPKM data, showing relative expression level of the target gene to the housekeeping gene (FPKM(TG/HKG)). TG: Target Gene; HKG: Housekeeping Gene. **b** Schematic representation of integration sites flanked by two genes. Chromosome information is provided on the right. **c** Examples of indels detected by deep sequencing. **d** Indel rates for each sgRNA detected by deep sequencing, with the respective highly expressed gene indicated below.

Next, we searched for genomic sites suitable for gene integration, aiming for locations that would facilitate efficient heterologous gene expression. We expected that the genomic regions supporting robust endogenous gene expression would be the open sites ideal for gene integration. To identify such sites, we analyzed an RNA-seq dataset^23^ reported previously and selected eight genes from the top 30 highly expressed genes (Fig. 2a). To avoid DNA fragment deletion between two cuts, these genes were distributed across five chromosomes, ensuring a significant distance between them even if located on the same chromosome. According to previous reports, the intergenic region flanked by two genes in the terminator-to-terminator orientation supports efficient heterologous gene expression^24^. Seven of the selected genes adhered to this orientation rule (Fig. 2b). Additionally, we included a gene locus (*HSP104*) that has been documented for its efficiency in supporting heterologous gene expression in the literature^19^.

Next, we designed sgRNAs to target these loci. To minimize off-target sites, we used an online tool named Cas-OFFinder^25^ to evaluate the specificity of each sgRNA. We designed two or three sgRNAs for each intergenic locus downstream of the selected genes, resulting in a total of 24 sgRNAs (sg1 − 24) (Fig. 2b, Supplementary Table 1). These sgRNAs contained at least two mismatches at their potential off-target sites (Supplementary Table 2). Two days after the transformation of the editing plasmids, genomic DNA was extracted for targeted deep sequencing. The deep sequencing results clearly indicated the occurrence of indels (Fig. 2c). Among them, six sgRNAs (sg 1, 4, 7, 8, 19, and 23) targeting five loci exhibited at least one sgRNA with an indel rate surpassing 80% (Fig. 2d). The potential off-target sites for these sgRNAs contained at 5-6 mismatches. Considering the sensitivity of SlugCas9-HF to the single mismatches, these sgRNAs are unlikely to cleave these potential off-target sites. These sgRNAs were subsequently employed for the gene integration experiments.

### Targeted integration with SlugCas9-HF

**Fig. 3:**
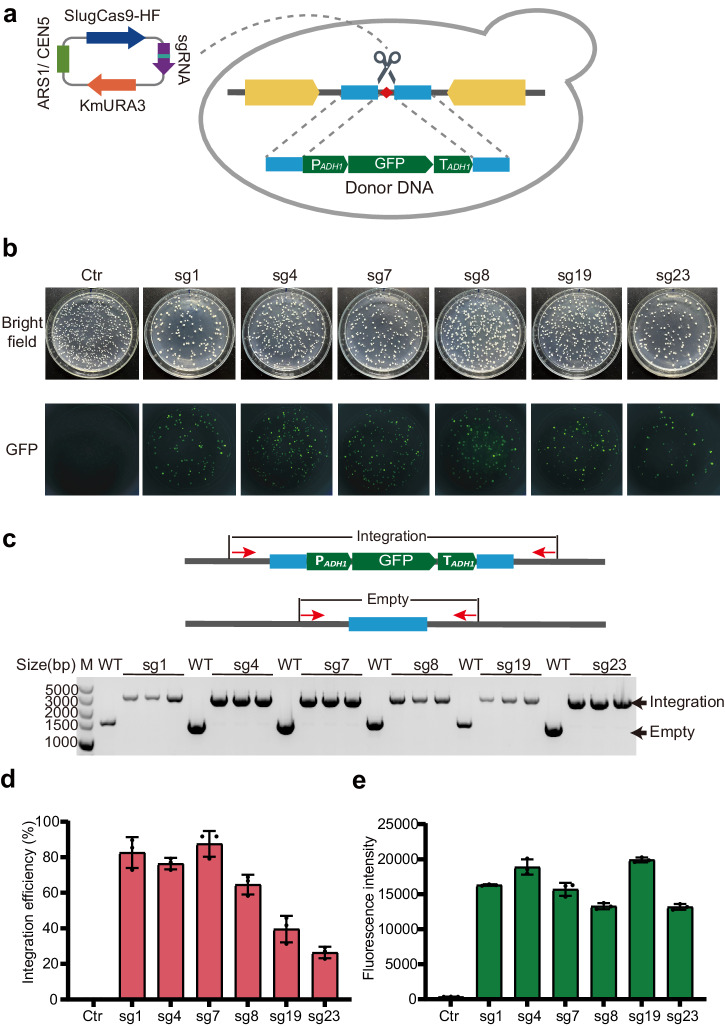
Test of gene integration. **a** Schematic illustration of SlugCas9-HF-mediated *GFP* gene integration via homologous recombination. **b** Images of yeast colonies expressing *GFP* post-genome editing. Green fluorescence in integrated yeast colonies was visible in the same visual field. Top: Bright field; Bottom: Fluorescent field. **c** A representative image of diagnostic PCR confirming gene integration into the target site. The schematic of the PCR design is shown above. Red arrows indicate primers. **d** Integration efficiency at selected integration sites. The efficiency for each site was evaluated by counting colonies with green fluorescence. Forty-eight colonies for each site were randomly streaked onto YPD medium for two days, as linear colonies’ green fluorescence was easier to distinguish. Three independent replicate experiments were performed. Error bars represent the mean ± SD of biological triplicates. **e** Impact of integration sites on the expression level of heterologous genes. The same expression cassette was integrated, and green fluorescence intensities were compared. Error bars represent the mean ± SD of biological triplicates.

To facilitate integrating exogenous genes into the genome, we established a CRISPR-SlugCas9-HF-based toolkit (Slug-toolkit). This toolkit contains SlugCas9-HF, a panel of sgRNAs, and a donor plasmid featuring 500 bp homology arms both upstream and downstream of the cutting site for each sgRNA. Users can insert genes of interest between the arms. We evaluated the efficiency of gene integration using the six selected sgRNAs. A *GFP* cassette, comprised of the *ADH1* promoter, *GFP*, and *ADH1* terminator, was inserted between the two homology arms (Fig. 3a). Crucially, the *GFP* cassette disrupted the Cas9 targeting sites on the donor, avoiding the donor plasmid from being cut. Two days after the transformation of the editing plasmids and PCR-amplified donor DNA fragments, *GFP* expression was observed for all tested sgRNAs (Fig. 3b). Conversely, when only the donor fragment DNA was transformed, no *GFP* signals were detected. These results indicated the Cas9-mediated stable integration of the *GFP* cassette into the genome.

To verify the integration of the *GFP* cassettes into the target sites, we designed primers flanking the homology arms. In the event of targeted integration, a larger PCR band would be observed. We tested three *GFP*-positive colonies for each sgRNA, and the PCR results consistently showed the presence of targeted gene integration in all colonies (Fig. 3c).

Next, we analyzed the integration efficiency for each sgRNA. For convenient *GFP* observation, we randomly selected colonies and streaked them onto the YPD solid medium for two days. The integration efficiency varied from 26% to 86%, with four sgRNAs exhibiting efficiency over 60% (Fig. 3d). To evaluate *GFP* expression levels, we picked up three *GFP*-positive colonies for each sgRNA and cultured them in 50 mL YD liquid medium for 72 h. *GFP* expression levels were measured using a fluorescence plate reader, revealing variations among the loci, with fluorescence intensity ranging from 13289 to 19902 (Fig. 3e). Notably, the sg19 locus exhibited the highest expression levels (19902). To evaluate the long-term gene expression potential, we cultured *GFP*-positive yeast (sg1 and sg7) in non-selective medium for up to 50 generations, and all cells maintained *GFP* expression (Supplementary Fig. 2), demonstrating the sustained expression capability of these loci.

**Fig. 4:**
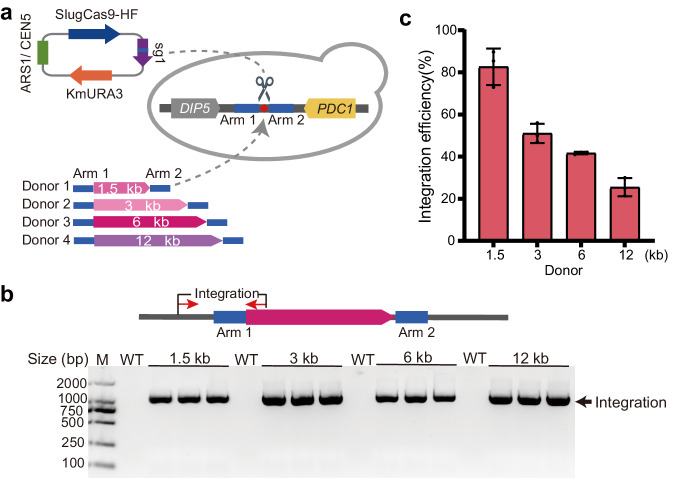
Evaluation of the ability to integrate heterologous genes of various lengths at a single site. **a** Schematic illustration of CRISPR-mediated gene integration via homologous recombination. Four gene fragment lengths (1.5 k, 3 k, 6 k, and 12 k) were selected and constructed as Donors. **b** A representative image of diagnostic PCR confirming gene integration. The schematic of PCR design is shown above. Red arrows indicate primers. **c** Integration efficiency of various lengths of heterologous genes at the sg1 locus. Integration efficiency was assessed by diagnostic PCR. Three independent replicate experiments were conducted. Forty-eight colonies for Donor- 1.5 kb and thirty- two colonies for Donor- 3 kb were randomly selected separately. For Donor- 6 kb and Donor- 12 kb, all colonies were selected due to the limited number of colonies formed. Error bars represent the mean ± SD of biological triplicates.

Next, we evaluated the influence of insertion fragment lengths on integration efficiency. For the sg1 locus, we designed four donor DNA fragments ranging from 1.5 kb to 12 kb (Fig. 4a). Two days after the transformation of editing plasmids and donor DNA fragments, integration events were detected by PCR for each colony (Fig. 4b). Integration events were observed for all donors, with efficiency decreasing as length increased (Fig. 4c). Specifically, the four donors (1.5 kb, 3 kb, 6 kb, and 12 kb) achieved integration efficiency of 83%, 51%, 42%, and 25%. Additionally, we investigated the influence of integrating a 12 kb fragment on yeast growth rate. The growth curves revealed that the 12 kb DNA fragment integration at this locus had no discernible impact on yeast growth (Supplementary Fig. 3).

We conducted additional tests to assess the efficiency of integrating GFP cassettes into the sg1 locus using shorter homology arms, measuring 50 bp and 200 bp in length (Supplementary Fig. 4). In comparison, the integration efficiency was found to be most favorable when employing the 500 bp homology arm. These findings indicated that SlugCas9-HF facilitated the integration of large DNA fragments.

### Multilocus gene integration with SlugCas9-HF in *K. marxianus*

**Fig. 5:**
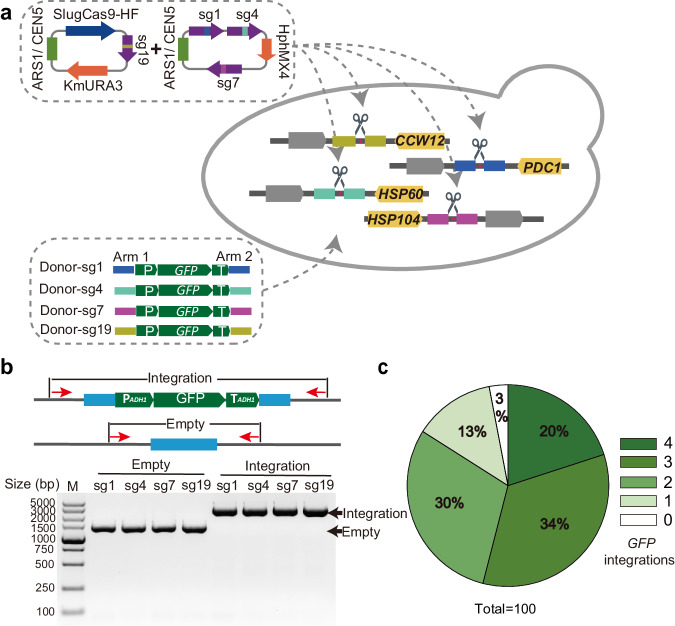
Establishment of a CRISPR/Cas9-mediated multilocus integration method in *K. marxianus.* **a** Diagram of the multiplex CRISPR-mediated integration system. This system includes SlugCas9-HF, a series of integration sites with corresponding sgRNAs, and donors containing promoters, target genes, terminators, and homology arms. It allows simultaneous integration of up to four genes. **b** A representative image of diagnostic PCR confirms gene integration into the four target sites. The left four lanes represent a yeast clone without integration, and the right four lanes represent a yeast clone with integration at four sites. The schematic of PCR design is shown above. Red arrows indicate primers. **c** Distribution of integration copy numbers within yeast clones. The pie chart is based on profiling 100 colonies.

The industrial applications of *K. marxianus* often necessitate the introduction of multiple genes involved in a biosynthetic pathway. We investigated whether multiple genes could be simultaneously integrated into these loci. Among the six sgRNAs tested in this study, sg23 was excluded due to its low integration efficiency, and sg8 was excluded because it targeted the same region as sg7. We focused on the remaining four sgRNAs (sg1, 4, 7, and 19). We assembled the three sgRNA expression cassettes (tRNAgly-sg1, SNR52-sg4, and tRNAgly-sg7) into one plasmid (LHZ1494, *HphMX4* marker conferring hygromycin resistance) (Fig. 5a). SlugCas9-HF and RPR1-tRNA^gly^-sg19 were expressed in another plasmid (LHZ1521, *URA3* marker). All four donors encoded a *GFP* cassette. After the cotransformation of sgRNA-expressing plasmids and donor DNA, transformants were incubated in a liquid SC-Ura medium with Hygromycin B for 24 h before being spread on the same culture medium plate. Two days later, we randomly picked 100 colonies to detect targeted integration of each sgRNA by PCR (Fig. 5b). The results revealed that 20% of colonies contained multilocus integration at four loci, 34% of colonies contained multilocus integration at three loci, and 30% of colonies contained multilocus integration at two loci (Fig. 5c). These data demonstrated that the Slug-toolkit enabled efficient simultaneous multigene integration.

Centromeric yeast plasmids can persist stably within cells for a long time. To facilitate the rapid elimination of plasmids expressing sgRNA and Cas9, cells harboring LHZ1493 (*URA3*) and LHZ1494 (*HphMX4*) were cultured in a non-selective medium overnight and subsequently plated onto 5-FOA plates, which are toxic to *URA3* cells. Colonies that emerged on the 5-FOA plates were transferred to a selective medium to evaluate the loss of both plasmids. The findings revealed that all clones had lost LHZ1493, with 98% also demonstrating loss of LHZ1494 (Supplementary Fig. 5). In conclusion, we have presented a method for efficiently eliminating plasmids from yeast cells.

### Application of the Slug-toolkit in the biosynthesis of heme

The Slug-toolkit allows the screening of the optimal combination of metabolic genes that can promote heme production in *K. marxianus*. Heme, a crucial cofactor for various functional proteins, plays an essential role in nearly all physiological processes of cellular life^26^. Two conserved biosynthesis pathways, the C4 pathway and C5 pathway, are involved in synthesizing the precursor of heme (5-aminolevulinate acid, 5-ALA) in nature^27,28^. Organisms like yeast, birds, mammals, and purple nonsulfur photosynthetic bacteria utilize the C4 pathway, where glycine and succinyl-coenzyme A (succinyl-CoA) are converted into 5-ALA by ALA synthase (Hem1)^29^. On the other hand, organisms like algae, plants, and bacteria such as *Escherichia coli* utilize the C5 pathway, where L-glutamate is used to generate 5-ALA catalyzed by glutamyl-tRNA synthetase (GltX), glutamyl-tRNA reductase (HemA), and glutamate-1-semialdehyde aminotransferase (HemL)^28,29^.

**Fig. 6:**
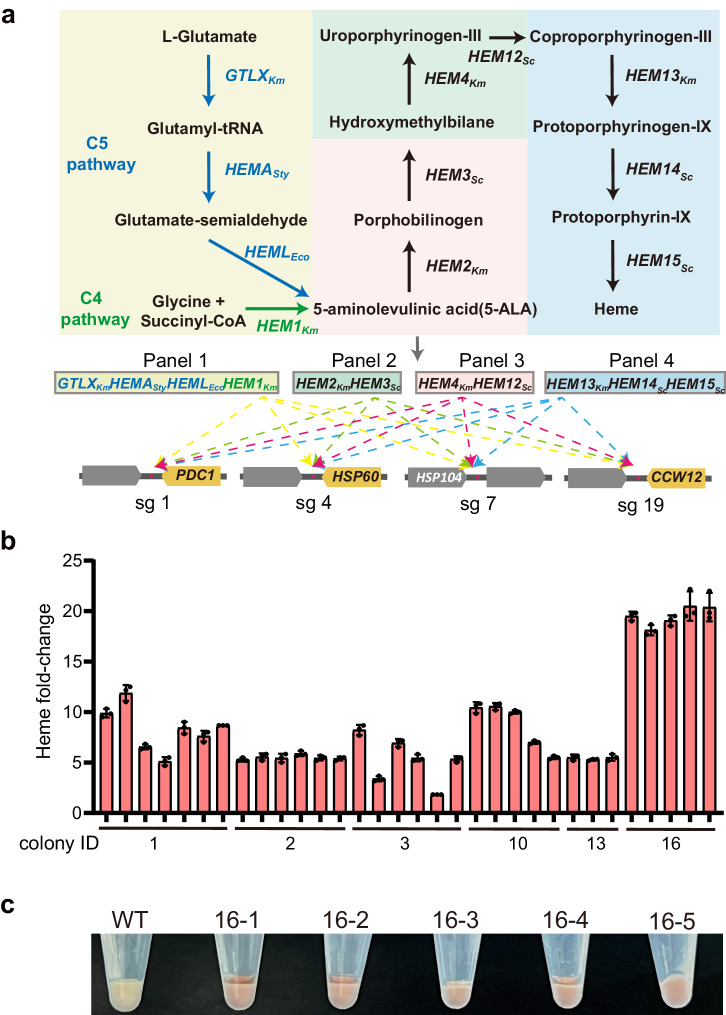
Application of the developed multilocus integration method in the biosynthesis of heme. **a** The reconstituted biosynthetic pathway of heme and its precursors. The key precursor of heme (5-ALA) can be accumulated by the C4 pathway (the encoding genes are shown in green) and the C5 pathway (the encoding genes are shown in blue). A total of 11 heterologous genes to be integrated were divided into four panels and ligated to construct four multiple-gene cassettes, which could be integrated into any of the four loci or none at one step. **b** Heme production of newly formed single colonies isolated from the 6 top production colonies in primary screening. Error bars represent the mean ± SD of biological triplicates. **c** Red pigment accumulation in the five most productive newly formed single colonies isolated from the mixed colony 16.

The conversion of 5-ALA to heme involves seven downstream enzymes: porphobilinogen synthase (Hem2), porphobilinogen deaminase (Hem3), uroporphyrinogen III synthase (Hem4), uroporphyrinogen III decarboxylase (Hem12), coproporphyrinogen III oxidase (Hem13), protoporphyrinogen oxidase (Hem14), and ferrochelatase (Hem15)^30^ (Fig. 6a). Metabolic engineering strategies, such as enhancing metabolic gene expression in the C5 pathway, have been applied to increase heme production in *E. coli*^31^.

To utilize both the C4 and C5 pathways for heme production, we overexpressed 11 genes catalyzing glycine, succinyl-CoA, and L-glutamate conversion into heme in *K. marxianus*. Based on our recent heme synthesis study^32^, we divided these genes into four panels (Fig. 6a).

Panel 1 included *K. marxianus HEM1*_*Km*_, *K. marxianus GTLX*_*Km*_, and two bacterial genes *HEMAS*_*ty*_, *HEML*_*Eco*_, resulting in an insertion fragment of 12,035 bp.

Panel 2 included *K. marxianus HEM2*_*Km*_ and *S. cerevisiae HEM3*_*Sc*_, resulting in an insertion fragment of 4641 bp.

Panel 3 included *K. marxianus HEM4*_*Km*_ and *S. cerevisiae HEM12*_*Sc*_, resulting in an insertion fragment of 5256 bp.

Panel 4 included *K. marxianus HEM13*_*Km*_, *S. cerevisiae HEM14*_*Sc*_, and *S. cerevisiae HEM15*_*Sc*_, resulting in an insertion fragment of 6988 bp.

Each of these genes was driven by a strong *K. marxianus* promoter^33^. Each panel of genes was flanked by homology arms up and downstream of sg1, sg4, sg7, and sg19 loci, resulting in a total of 16 donor vectors. This design facilitated the integration of each gene panel into any of the four loci, thereby enabling the examination of potential multi-copy effects of a specific gene panel on heme production.

The donor and sgRNA-expressing plasmids were combined and cotransformed into yeast. Following two-and-a-half-hour incubation in the liquid medium, yeast cells were plated on a solid medium to allow colony formation. A total of 624 potential integration combinations were anticipated through a single transformation targeting the four loci. We obtained a total of over 2000 transformants across 20 solid medium plates. To identify strains with high heme accumulation, indicated by a red color due to heme bound with iron ions (Supplementary Fig. 6), 49 colonies displaying a strong red color were selected for primary screening. Heme production in these colonies was measured, revealing a fold-change ranging from 0.8 to 6 compared to unmodified *K. marxianus* (Supplementary Fig. 7a).

To further analyze the top producers, the six colonies with the highest heme production were chosen for genotyping. However, PCR amplification results indicated that some colonies had multiple heme panels integrated into the same locus (Supplementary Fig. 7b), indicating a mixed colony. To address this, the top six production colonies (colony ID: 1, 2, 3, 10, 13, and 16) were spread to form new single colonies. From each colony, 3 to 7 newly formed colonies with strong red colouration were selected, and their heme production was measured. In comparison to unmodified *K. marxianus*, the fold-change in heme production ranged from 2 to 20 (Fig. 6b). The top five heme production colonies were all derived from the original colony #16 (Fig. 6c). Genotyping of these five colonies revealed the insertion of three copies of panel 3 into the sites of sg1, sg7 and sg9 (Supplementary Fig. 8a, b), suggesting a substantial contribution of high copies of *HEM4*_*Km*_ and *HEM12*_*Sc*_ to the improvement of heme production.

## Discussion

The identification of genomic loci supporting long-term and robust heterologous gene expression in *K. marxianus* is a significant advancement that enhances its potential as a cell factory for industrial applications. Unlike other well-studied yeast species like *S. cerevisiae*, where specific loci are routinely employed for gene expression^34,35^, the unique genomic landscape of *K. marxianus* required dedicated exploration. In this study, we successfully identified four specific loci within intergenic regions of the *K. marxianus* genome that support robust exogenous gene expression without disrupting endogenous gene functions. This finding is crucial for the construction of cell factories, as it provides a foundation for stable and sustained expression of desired genes over extended periods.

Moreover, the development of toolkits facilitating efficient and multilocus gene integration into these identified loci is a key innovation. Previous studies have demonstrated the feasibility of integrating a DNA fragment containing multiple genes into a single locus^18^. As an extension, we demonstrated the feasibility of integrating genes into multiple sites simultaneously. These toolkits streamline the process of genetic manipulation in *K. marxianus*, enabling researchers and industrial practitioners to engineer strains tailored for specific applications. The ability to integrate multiple genes into these loci is particularly advantageous for constructing complex metabolic pathways or enhancing the expression of multiple genes simultaneously.

While SpCas9 has demonstrated its utility for gene deletion and gene integration in *K. marxianus*^9,21^, the occurrence of nonnegligible off-target mutagenesis has raised concerns^36^. Off-target effects can potentially limit the broader use of SpCas9 for precise biosynthesis applications, which can be partially avoided from the primer design but still requires further improvement in the specificity of the Cas9 protein since off-target editing might not only reduce the production of host cells but also potentially damage their function. In response to the limitations of SpCas9, our study introduces SlugCas9-HF^20^ as an alternative Cas9 variant for genome editing in *K. marxianus*. SlugCas9-HF does not tolerate single mismatches and exhibits higher specificity than SpCas9 in *K. marxianus*. This advancement addresses concerns associated with off-target effects, thereby expanding the utility of CRISPR-Cas9 technology for precise and reliable genome editing in industrial applications and synthetic biology.

## Materials and methods

### Strains and media

*URA3* was deleted in Wild-type *K. marxianus* strain FIM-1 (China General Microbiological Culture Collection Center, CGMCC No. 10621) to obtain FIM-1∆U^23^. T1 was a mutant strain originating from FIM-1∆U^37^. No NHEJ-related mutations have been found in the ORFs of T1 (Supplementary Table 3), which was selected as the host cell for this study.

*K. marxianus* cells were cultivated at 30°. YPD medium (2% w/v peptone, 2% w/v glucose, 1% w/v yeast extract, 2% w/v agar for plates). Synthetic dropout media without uracil (SC-Ura) medium^38^. YPD or SC medium was supplemented with 200 mg/mL Hygromycin (H8080, Solarbio, Beijing, China) as indicated.

### SgRNAs design

An online tool called Cas-OFFinder was used to evaluate the specificity of each sgRNA in designing sgRNAs targeting editing sites. Cas-OFFinder is not limited by the number of mismatches, so all of the possible mismatches of the sgRNA in the genome were predicted, from which the sgRNA with the lowest possibility of off-target sites was selected.

### Plasmid construction

Plasmids used in this study are listed in Supplementary Table 4. Primers used are listed in Supplementary Table 5.

For the construction of SlugCas9-HF expression plasmid, the SpCas9 and its matching sgRNA scaffold in CRISPR vector LHZ531^39^ were replaced by SlugCas9-HF and Sa sgRNA scaffold: *ARS1/CEN5* backbone of LHZ531 was PCR-amplified with primers New-1-F and CPSlugCas9-ARS1-R; fragment of SlugCas9-HF on plasmid SlugCas9-HF was firstly performed synonymous mutation using primers SyM-A-F/ SyM-A-R in order to use BspQ I for adding sgRNA, and then was amplified from the plasmid by primers SlugCas9-ARS1-F/SlugCas9-ARS1-R; Sa sgRNA scaffold was amplified from plasmid SlugCas9-HF by primers CPWhole-F/CPWhole-R. These fragments were recombined using the NEBuilder HiFi DNA Assembly Cloning Kit [New England Biolabs (NEB), E5520] to generate LHZ1493, the CRISPR vector of SlugCas9-HF for genome editing in *K. marxianus*. To construct an empty vector plasmid without SlugCas9-HF protein (LHZ1561), we performed PCR-amplified with primers emptyCas9-F/ emptyCas9-R and connected it using T4 DNA Ligase (NEB). To construct CRISPR plasmids (LHZ1495 ~ 1554) used in editing different target sites or mismatch sites in this study, primers containing 21 bp (for SlugCas9-HF) or 20 bp (for SpCas9) target sequences were annealed in pairs to generate sgRNAs, which were inserted into the LHZ1493 or LHZ531 plasmid between two BspQ I restriction sites.

The integration donor plasmids (LHZ1555 ~ 1560) were constructed by cloning the upstream (~500 bp) and downstream (~500 bp) homology arm sequences into pUC19 (WeiDi) backbone (PCR-amplified with primers HP-pUC19-F/HP-pUC19-R) using the NEBuilder HiFi DNA Assembly Cloning Kit. The *GFP* gene flanked by *ADH1* promoter and terminator (amplified from the genome of FIM1∆U), or any other gene cassettes to be integrated into the genome, were inserted between the upstream and downstream homology arm using the NEBuilder HiFi DNA Assembly Cloning Kit, after which the corresponding donors were released by PCR amplification with sgXu-F/sgXd-R primers.

To construct the triple sgRNA expression plasmid (LHZ1494) for multiplex genome editing, 13-Myc-*KanMX6* in pFA6a-13Myc-KanMX6^40^ was replaced by a *KmARS1/CEN5*-*HPHMX4* cassette which generated a plasmid backbone. Three sgRNA expression cassettes (tRNAgly-sg1, SNR52-sg4, and tRNAgly-sg7) were cloned into the plasmid backbone using the NEBuilder HiFi DNA Assembly Cloning Kit. Among them, tRNAgly-sg1 and tRNAgly-sg7 cassettes were PCR-amplified from the corresponding CRISPR plasmids (LHZ1503 and LHZ1503), while the SNR52-sg4 cassette was first constructed on pRS425-Cas9-2xSapI through two BspQ I restriction sites and then PCR-amplified. Full sequences of LHZ1493, LHZ1494 and pRS425-Cas9-2xSapI were listed in Supplementary Data 2.

### Analysis of RNAseq dataset

Previously reported RNA-seq datasets (Bioproject Accession: PRJNA658204) were subjected to analysis^23^. The top 30 *K. marxianus* genes with the highest relative expression level to the housekeeping gene (FPKM(TG/HKG)) after 24 h of culturing in YPD were selected.

### Targeted deep sequencing

Targeted deep sequencing, also known as targeted next-generation sequencing (NGS), is a high-throughput sequencing method used to analyze specific regions of interest within a genome. Unlike whole-genome sequencing, which sequences the entire genome, targeted deep sequencing focuses only on particular genomic regions where Cas9 generated indels. We used PCR to amplify the target sites and applied NovaS4 sequencing platforms provided by Mingma Technologies to sequence the PCR products. Typically, over 300,000 reads were obtained for a locus. Genome assembly ASM185444v2 serves as the reference genome.

### Determination of CRISPR efficiency

To perform gene editing or/and gene integration at target sites, the CRISPR plasmids were transformed or cotransformed with the corresponding linear donors into T1 by a lithium acetate method^41^. Transformants were selected on SC media without uracil (SC-Ura) plates after being incubated at 30 °C for 1–2 days.

To measure the genome editing efficiency, the genomic DNA of transformants was isolated by a TIANamp Yeast DNA Kit (TIANGEN), and the target sites were PCR-amplified and purified by a Gel Extraction Kit (QIAGEN) for targeted deep sequencing.

To measure gene (*GFP*) integration efficiency in the six selected integration sites, forty-eight colonies for each site were randomly streaked onto the YPD medium for two days since the green fluorescence of linear colonies was easier to distinguish. Three independent replicate experiments were performed. The integration efficiency was calculated as the percentage of fluorescent colonies.

For the determination of multilocus genome integration efficiency, the plasmid LHZ1494 and LHZ1521 were cotransformed with all of the four corresponding linear donors carrying a *GFP* gene cassette. Transformants were incubated in liquid SC-Ura medium with Hygromycin B for 24 h before spread on the same culture medium plate. Two days later, 100 colonies were randomly picked to detect the targeted integration of each sgRNA site by colony PCR. The integration efficiency was calculated as the percentage of colonies with all PCR-positive amplicons.

### Stability of the *GFP*-integrated yeast

The *GFP*-positive strains were grown in YPD medium overnight, and the culture was inoculated into fresh 50 mL YPD medium to start at an OD_600_ of 0.2. Cells were grown for 48 h (about seven generations). This process was repeated for 14 consecutive days (approximately 50 generations). The diluted cultures collected after indicated generations were spread on YPD plates, which were incubated at 30 °C for 1–2 days. The stability of *GFP*-integrated yeast was determined by observing green fluorescence under blue excitation light.

### Plasmid loss and detection

Yeast cells containing the two plasmids of our Slug-toolkit, LHZ1493 (*URA3*) and LHZ1494 (*HphMX4*), were grown in the YPD medium overnight. The culture was grown to an OD_600_ of about ten and then diluted 1000-fold. A total of 100 μL of the dilution was spread onto YPD plates containing 5-fluoroorotic acid (5-FOA), which is lethal to *URA3* cells. Fifty clones that grew on the 5-FOA plates were randomly selected and transferred onto YPD, SC-Ura and YPD+ Hygromycin plates to assess the loss of the two plasmids. The experiment was repeated three times.

### Measurement of Heme and *GFP* gene expression

Cells were grown in 50 mL YD medium (4% w/v glucose, 2% w/v yeast extract) in a shake flask for 72 h. For heme, the total content of heme and free porphyrin in the cells was measured by a fluorescence-based method as described before^42^. Cells were collected at OD_600_ ∗ mL = 8. After discarding the supernatant, the remaining cells were resuspended in 500 μL of 20 mM oxalic acid and then kept in dark at 4 °C overnight. 500 μL of preheated 2 M oxalic acid was added to the mixture, which was then transferred into an amber tube and heated up to 95–98 °C for 30 min. 200 μL of clear supernatant was then moved to a black 96 plate and analyzed for fluorescence with excitation at λ = 400 nm and emission at λ = 600 nm, using a fluorescence plate reader (Bio Tek Eon (USA), H1202488).

For measuring *GFP* expression levels, cultures after 72 h of incubation were shaken and mixed well, 100 μL of the culture was collected, and the green fluorescence intensities were measured at 485–528 nm.

### Statistics and reproducibility

Microsoft Office 365 Excel and GraphPad Prism 5 were used for data averaging, calculation of standard deviations, and evaluation for statistical significance. All the measurements were taken from distinct samples in at least three independent biological repeats unless otherwise stated. Data were expressed as mean ± SD. Average is shown in bar graph as well as individual data points. All source data underlying the main figures are available in Supplementary Data 1. The sample sizes and number of repeats (n) are described or defined in the figure legends and Supplementary Data 1 in each particular case.

### Reporting summary

Further information on research design is available in the Nature Portfolio Reporting Summary linked to this article.

### Supplementary information

Supplementary information

Description of Additional Supplementary Files

Supplementary Data 1

Supplementary Data 2

Reporting Summary

## Data Availability

The RNA-seq datasets being subjected to analysis in this study can be found in online NCBI Sequence Read Archive (SRA) repositories as follows: https://ncbi.nlm.nih.gov/bioproject/658204. All data generated during the study are available within the article and its Supplementary Information files. Source data are provided in Supplementary Data 1. Uncropped and unedited blot/gel images were provided in Supplementary Information files (Supplementary Fig. 9-12). Full sequences of plasmids LHZ1493, LHZ1494 and pRS425-Cas9-2xSapI are provided in Supplementary data 2, and plasmids LHZ1493, LHZ1494 will be available via the non‐profit plasmid repository Addgene (Plasmid ID: 222102 and 222104). Any remaining information can be obtained from the corresponding author upon reasonable request.
