# Supplementary material for: Efficient and markerless gene integration with SlugCas9-HF in Kluyveromyces marxianus
Source: Commun Biol. 2024 Jul 2;7:797. doi: 10.1038/s42003-024-06487-w (PMC11219867; doi:10.1038/s42003-024-06487-w)
Supplement: Supplementary file 1 — Supplementary information [file 42003_2024_6487_MOESM1_ESM.pdf]

Efficient and markerless gene integration with SlugCas9-HF in *Kluyveromyces marxianus*

Huanyu Zhou, Tian Tian, Hong Lu, Yao Yu, Yongming Wang

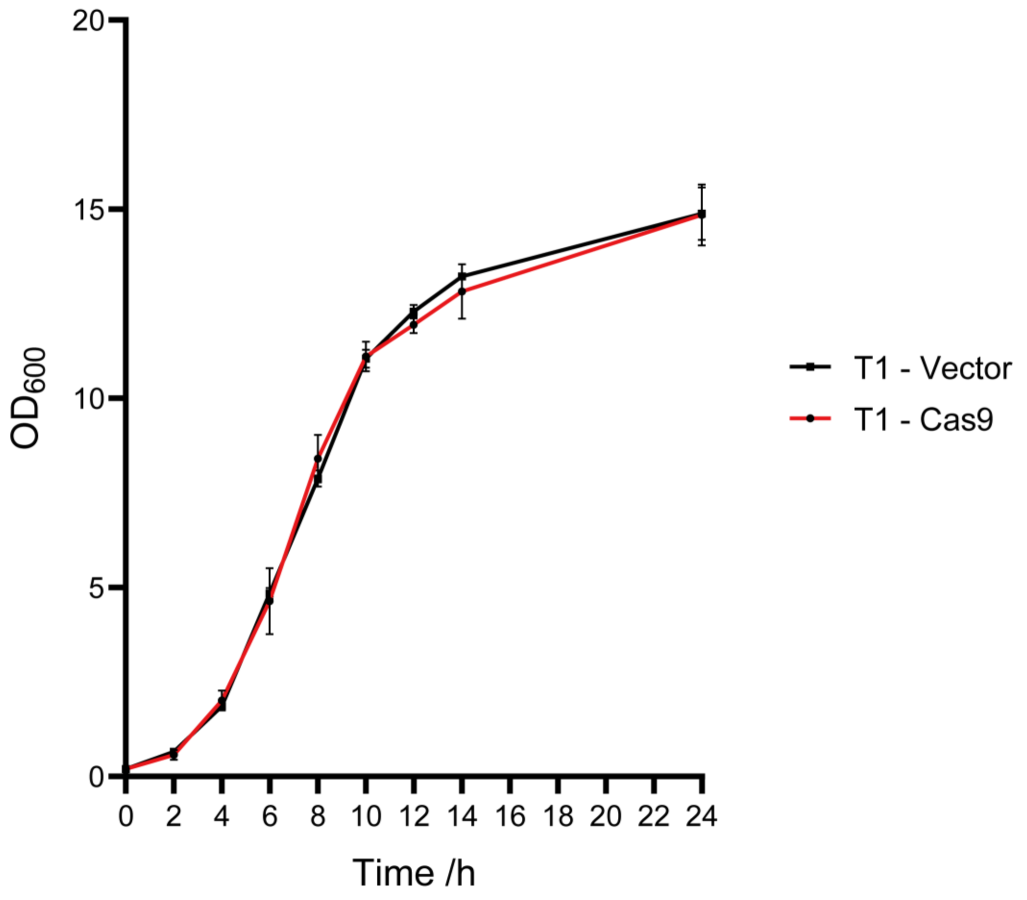

Supplementary Figure 1. The effect of Cas9 expression on the growth of *K. marxianus*. The overnight culture of T1 cells containing the vector (LHZ1561) or a plasmid expressing SlugCas9-HF (LHZ1493) was diluted into SC-Ura medium to an initial OD_{600} of 0.2 and grown at 30 degrees. OD_{600} was measured every 2 hours over 24 hours. Error bars represent the mean ± SD of biological triplicates.

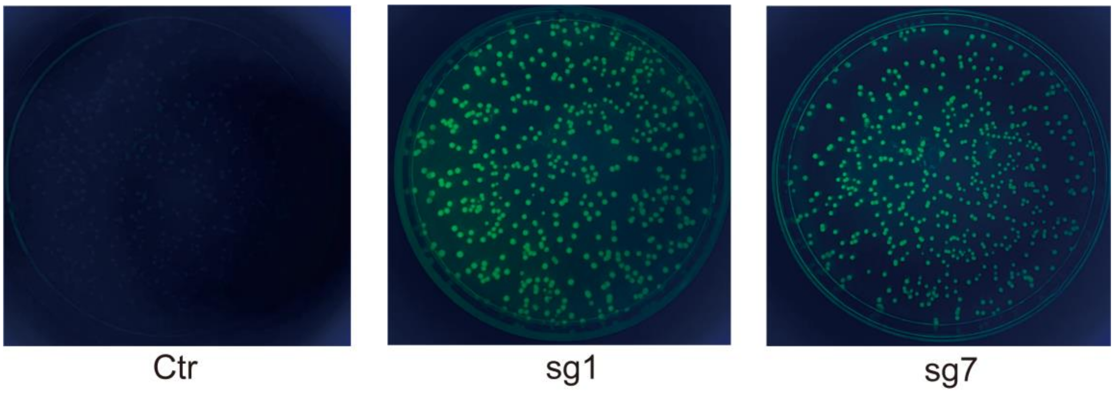

Supplementary Figure 2. Stability of the *GFP* expression. Strains were cultured in the non-selective medium for ~50 generations (via serial seven transfers) and then spread into YPD agar plates. The presence of green fluorescence in all colonies indicated high stability of the *GFP* integration. Yeast cells without *GFP* integration were included as a negative control.

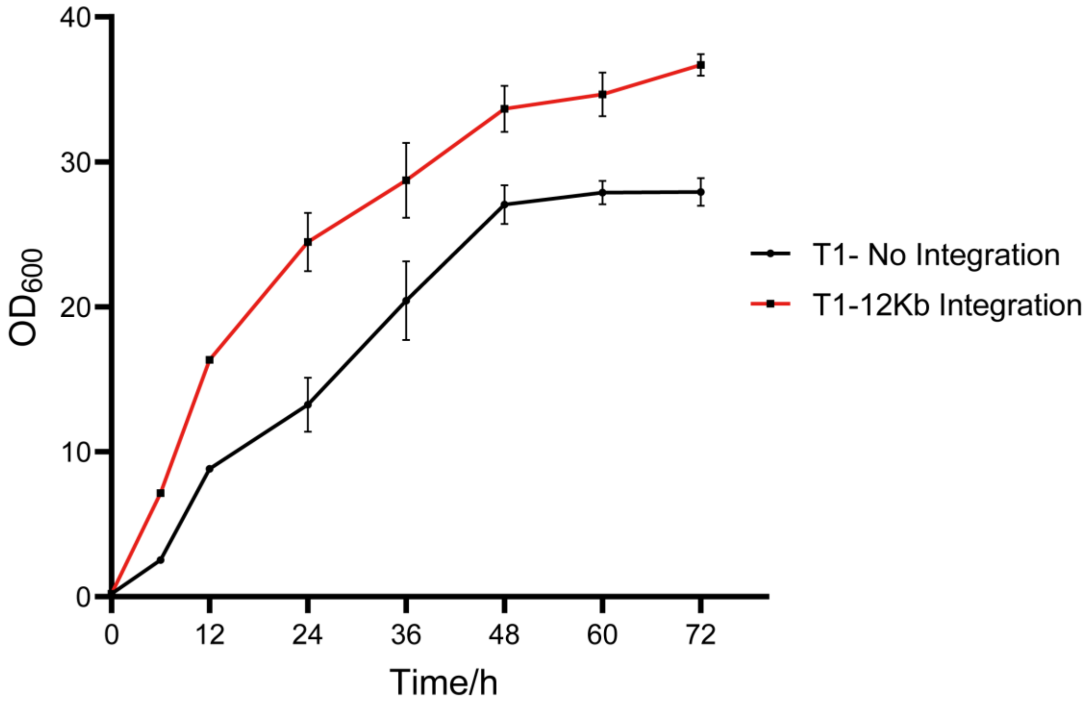

Supplementary Figure 3. The effect of a 12kb gene integration on the growth of *K. marxianus*. T1 strains without gene integration were used as a negative control. Three colonies were randomly selected and cultivated in non-selective medium YD. OD_{600} was measured every 6 hours or 12 hours to obtain the growth curves. Error bars represent the mean ± SD of biological triplicates.

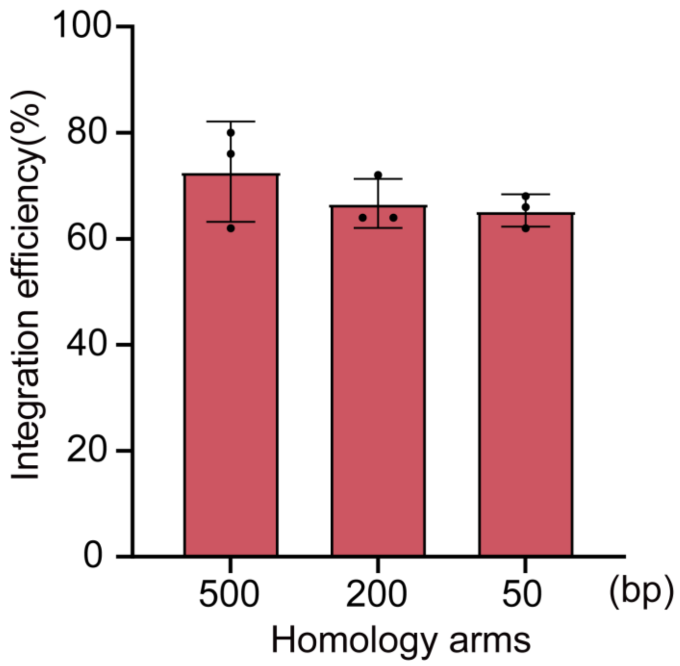

Supplementary Figure 4. The efficiency of integrating *GFP* cassettes (1415 bp) with shorter homology arms. The efficiency was evaluated by counting colonies with green fluorescence. Fifty colonies for each site were randomly streaked onto the YPD medium for two days for easier statistics. Three independent replicate experiments were conducted. Error bars represent the mean ± SD of biological triplicates.

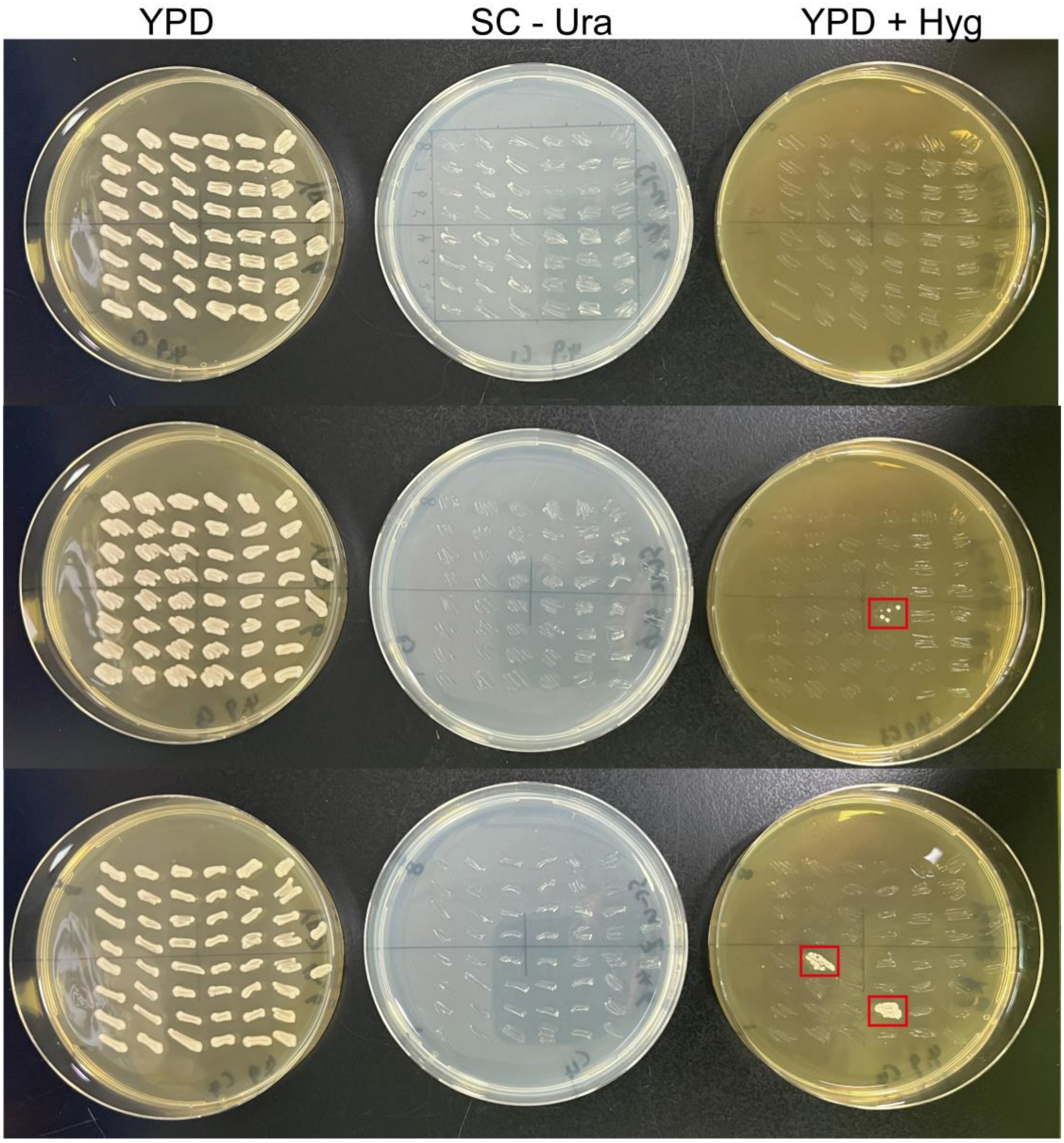

Supplementary Figure 5. Assess the loss of Slug-toolkit plasmids. Cells containing Slug-toolkit plasmids were growing in the non-selective YPD overnight and spread onto 5-FOA plates. Fifty colonies were randomly selected from the 5-FOA plates and transferred onto YPD, SC-Ura and YPD+Hygromycin (Hyg) plates. The experiment was repeated three times, and representative pictures of the plates were shown. The colonies in red boxes contained LHZ1494 (HphMX4).

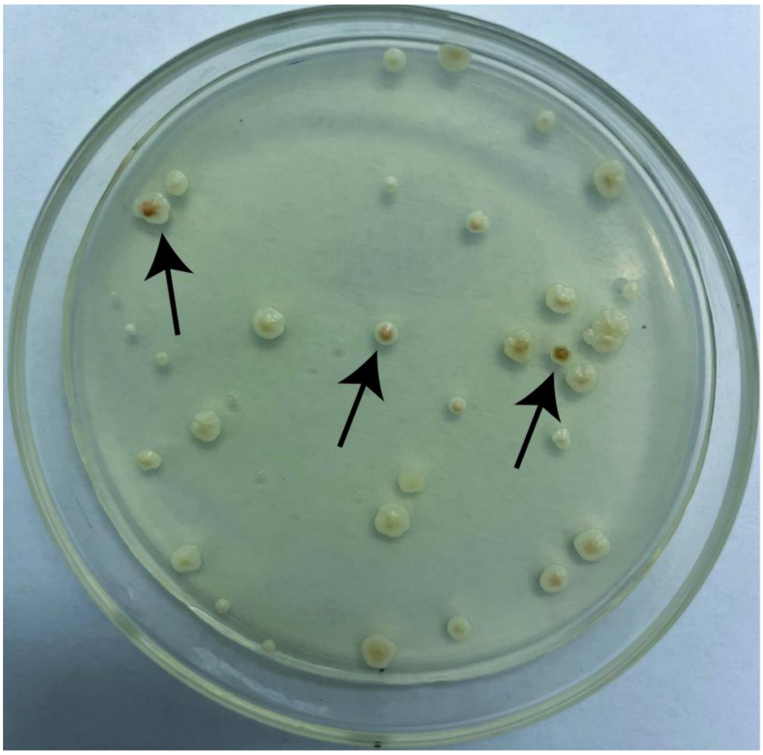

Supplementary Figure 6. An example of *K. marxianus* colonies displaying accumulated heme. Colonies with red pigment accumulation due to ferrous ions are indicated by arrows.

a

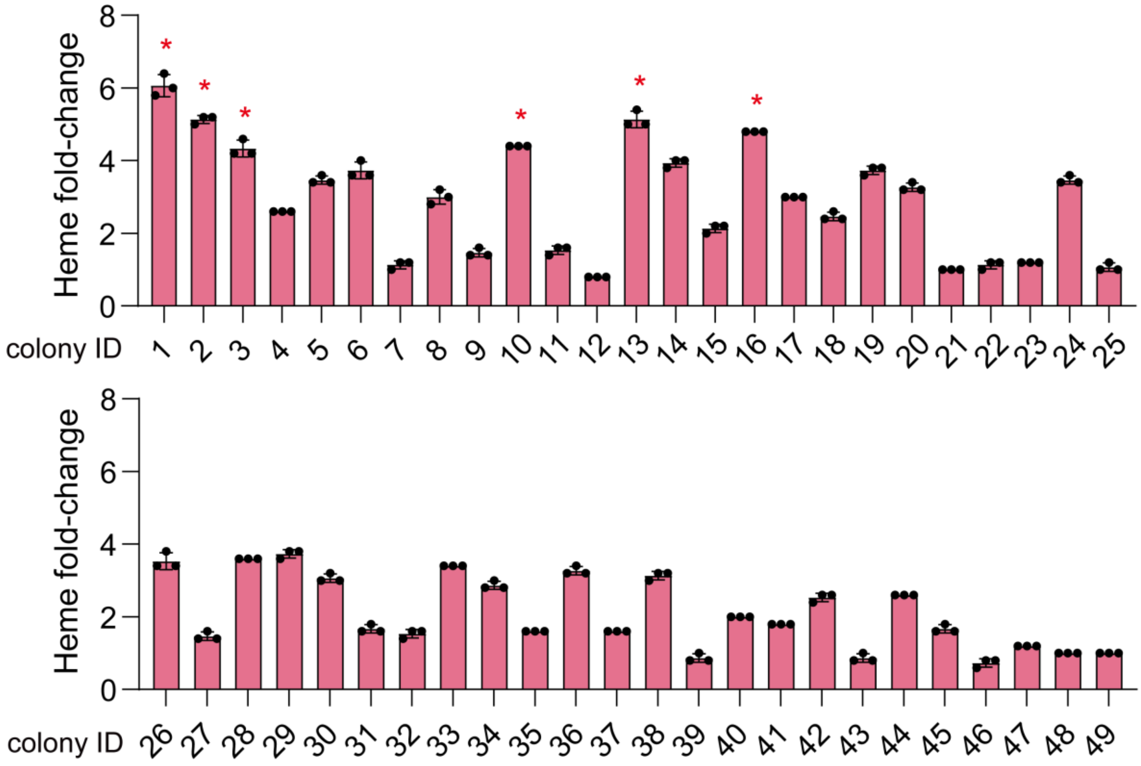

b

	1	2	3	10	13	16
sg1	Panel 4	Panel 3	Panel 2	Panel 2, 3	Panel 2	Panel 3
sg4	Panel 4	/	/	/	/	/
sg7	Panel 1	Panel 1	Panel 1, 3	Panel 1	/	Panel 1, 3
sg19	Panel 1	Panel 2	Panel 2, 3	Panel 2, 3	Panel 2, 4	Panel 3

Supplementary Figure 7. Heme production analysis of 49 colonies. (a) Forty-nine colonies displaying a strong red colour were selected for primary screening and assessed for heme production. The top six colonies with the highest heme production are indicated with red asterisks. Error bars represent the mean ± SD of biological triplicates. (b) Genotyping was performed on the six colonies with the highest heme production. The colony ID is shown on the top. Some colonies show multiple DNA fragments at the same locus, indicating potential mixing in the colony.

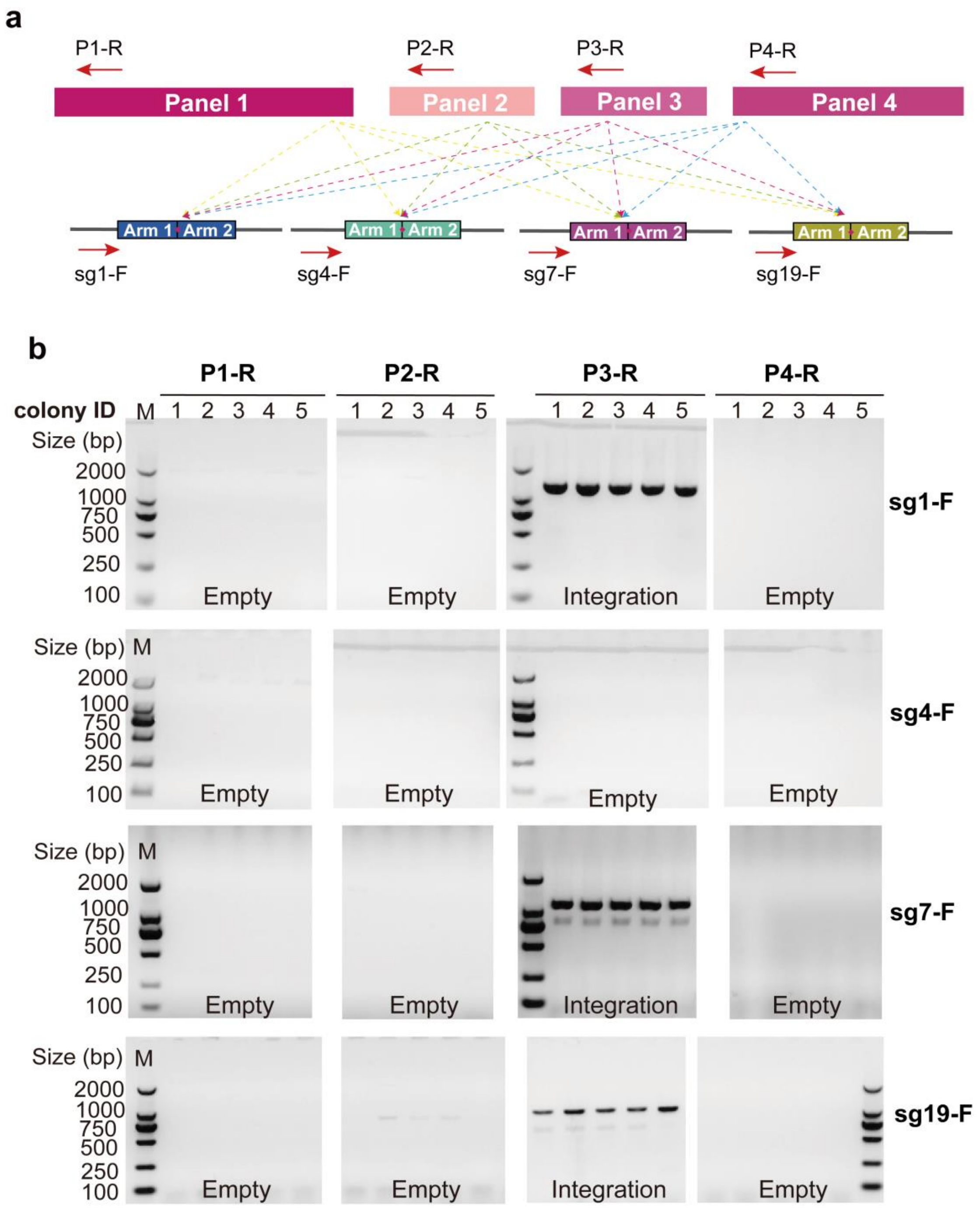

Supplementary Figure 8. Verification of gene integration in top five heme production colonies derived from the original colony #16. (a) Schematic of diagnostic PCR to detect the gene integration into the four target sites. (b) Images of diagnostic PCR detecting gene integration into the genome. The forward and reverse primers used are displayed on the right and upper sides, respectively.

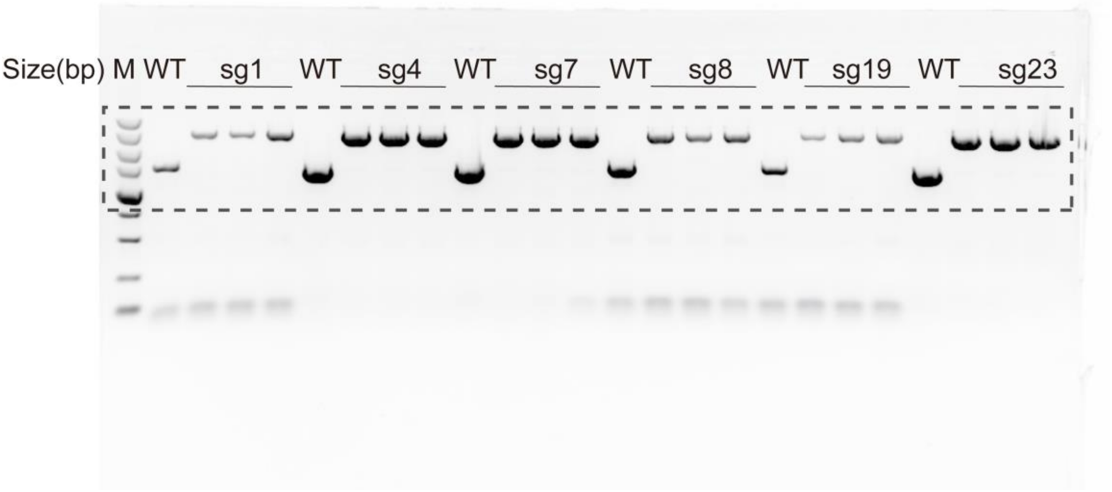

Supplementary Figure 9, related to Figure 3c. Un-cropped gels and blots. Panels labeled as in Figure 3c.

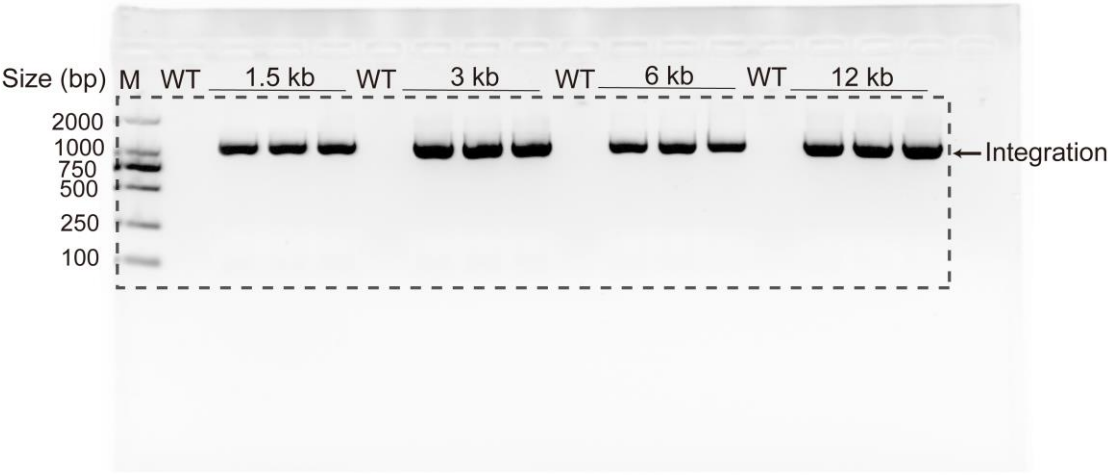

Supplementary Figure 10, related to Figure 4b. Un-cropped gels and blots. Panels labeled as in Figure 4b.

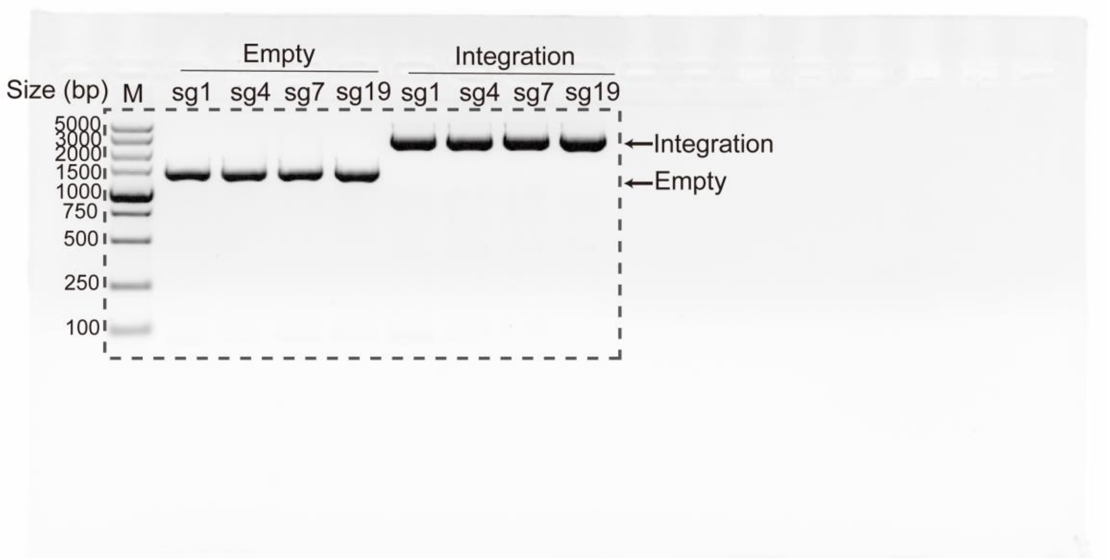

Supplementary Figure 11, related to Figure 5b. Un-cropped gels and blots. Panels labeled as in Figure 5b.

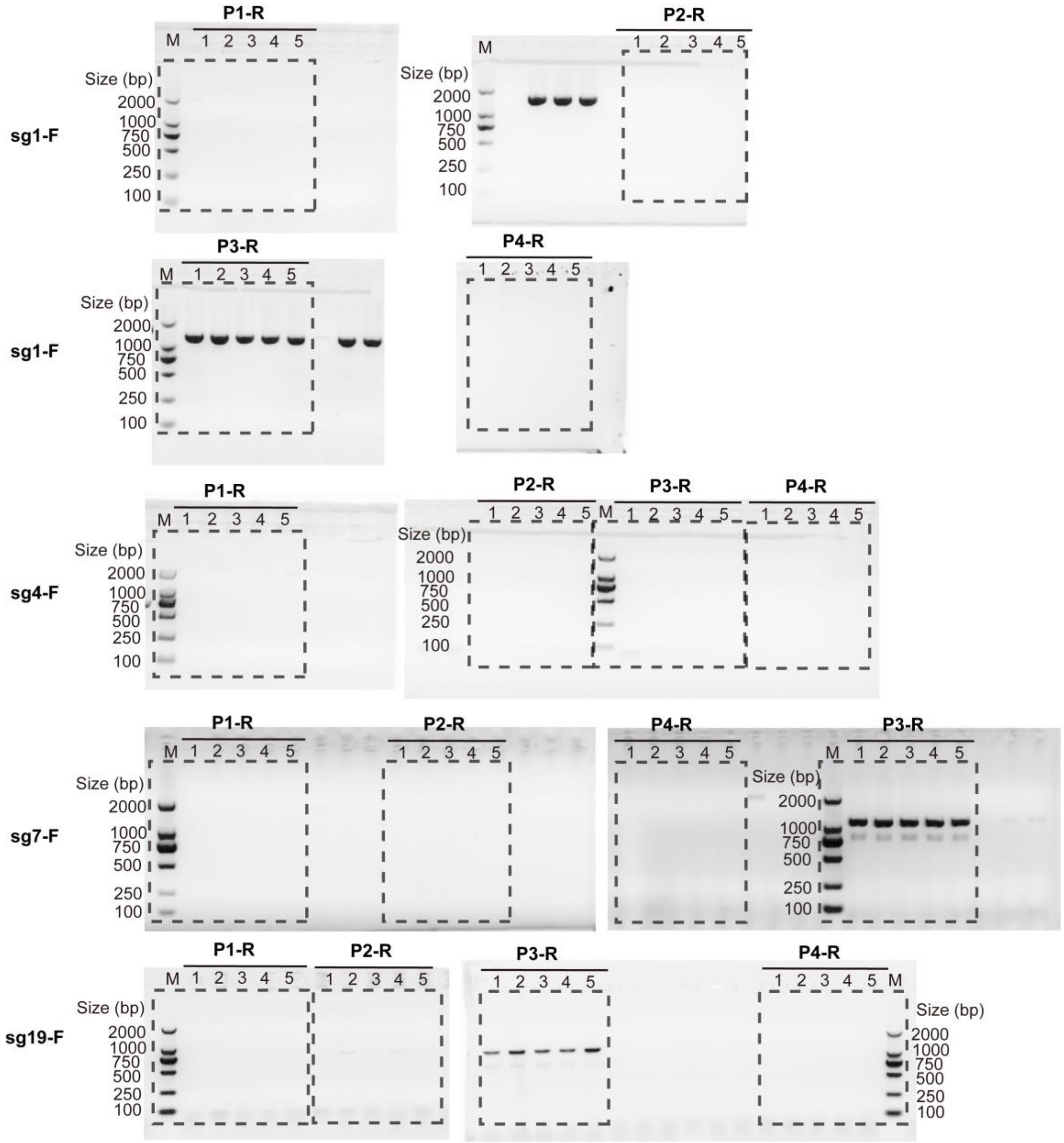

Supplementary Figure 12, related to Supplementary Figure 8. Un-cropped gels and blots. Panels labeled as in Supplementary Figure 8.

Supplementary Table 1. Sites identified potential for integration.

Sites	gRNA sequence	position
sg1	aattgggggaattataagcgt	CP015059.1 (1,020,628..1,020,648)
sg2	aagttacaagttacaagttac	CP015059.1 (1,020,585..1,020,605)
sg3	tatgccaaataaagtaaaagg	CP015059.1 (1,020,430..1,020,450)
sg4	tagaaaaacagtagtggaagg	CP015055.1 (601,263..601,283)
sg5	aaaaggcatctctatttacac	CP015055.1 (601,390..601,410)
sg6	atcctcaatcagatactcatt	CP015057.1 (232,097..232,117)
sg7	gtacacagcattggaaatacc	CP015057.1 (232,396..232,416)
sg8	agtttccgagggaggttgccg	CP015057.1 (232,469..232,489)
sg9	agagaactggaaaaatgtgag	CP015058.1 (532,242..532,262)
sg10	gaacgaactgagaattatcgt	CP015058.1 (532,293..532,313)
sg11	ataaatgaatgacgttacgag	CP015058.1 (532,681..532,701)
sg12	gaaaggaaaagggaaacggaa	CP015054.1 (658,358..658,378)
sg13	cacatgataaagatatagtgc	CP015054.1 (658,322..658,342)
sg14	tatataaaatgtcgctgtgac	CP015054.1 (658,224..658,244)
sg15	aagggttaacaactaactgac	CP015057.1 (525,326..525,346)
sg16	ttcaaatgttaatggaagtgg	CP015057.1 (525,640..525,660)
sg17	ggcataaaaaaagacaaggca	CP015057.1 (525,664..525,684)
sg18	gggaaaattcgaccaaaacgg	CP015057.1 (423,657..423,677)
sg19	gggatgagcgaaacgaaacgt	CP015057.1 (423,718..423,738)
sg20	tttgtgtgtgaacgtacatca	CP015057.1 (423,773..423,793)
sg21	tataaaaagcgtcagagcgct	CP015059.1 (341,803..341,823)
sg22	ctcgtaacaacaaggggaagg	CP015059.1 (341,696..341,716)
sg23	ataagtagtattgagagctga	CP015054.1 (1,242,153..1,242,173)
sg24	ccctggaatatgaaggaagcg	CP015054.1 (1,241,892..1,241,912)

Supplementary Table 2. Minimum mismatches of sgRNAs at potential off-target sites. Mismatched bases are indicated by lowercase letters.

Sites	Minimum mismatches	Number of off-target sites with minimum mismatches	Sequence of off-target sites
sg1	5	1	AATTGGGaGgATTtgAAGaGTGTGG
sg2	3	1	AAGTcACAAGTcACAAGTcACGTGG
sg3	5	7	atTGCCAAATAcAGTAAAAacGTGG
			TAactCAAgTAAAGTAAtAGGTTGG
			TATGCCtgATAtcGTtAAAGGAAGG
			aATGCaAAAgAAAGaAAgAGGTGGG
			TATGCCAAATcgAaTAtAAaGATGG
			TtgGCCAAATcAAGaAgAAGGTTGG
			aATGtCAgAaAAAGTAAgAGGTAGG
sg4	5	2	TAGAAgAACAGTAGTtagtGGGGGG
			aAaAAAAAaAagAGTGGAAGGCTGG
sg5	5	3	AAAAGGttTaTCTtTTTACAtTGGG
			AAAtGaCgcaTCTATTTACACTAGG
			AAAAGGgtTCTCTAgTTAtAgACGG
sg6	5	2	gaCgTCAgTCAGATAtTCATTGAGG
			ATCgTCgggCAGATcCTCATTCCGG
sg7	5	1	aTACACAcacTTGGAAATcCCAGGG
sg8	6	13	AGTTTgCGAtGGAatTTGgCcCTGG
			gGTaTtCGAGGGAaGgTcCCGCGGG
			AaTTaCCGAcGGAGcTTGggGAAGG
			AtTaTCgGgaGGAtGTTGCCGGAGG
			AGTTTCCtgGGGgtGTTtCCcCGGG
			AcTggCCGAtGGtGGTgGCCGCTGG
			AagTaaCGAGGGAaGTTGaCGAAGG
			AcaTTagGAaGGAGGcTGCCGGAGG
			AGgTTCCctGGGgGGTTcCCcCAGG
			gGgTTCCctGGGAGGTTcCCtGGGG
			AGTcTgCGAcGaAacTTGCCGTTGG
			AaTTTCCGAGGaAGtTgGatGCTGG
			AGTcaCCGAaGGAGGcaGCtGGTGG
sg9	5	6	ctAGtACTGGAAAcAaGTGAGCAGG
			AGAcAACTtGgAAAATtTaAGGTGG
			AGAGAcCTGGAtcAATGgGAtCTGG
			gaAaAACTGaAAAAtTGTGAGGCGG
			AGAaAAaTaGAAAAATGgaAGTAGG

			AGAGAAgTGGAAgAATacGAaAGGG
sg10	6	7	GAACtAAtTaAGAtTTATtGgTTGG
			GgAtGAAaTcAGAAcTATtGTCAGG
			GcACGAACgGAcAATTTtTgGgTTGG
			cAACGAACTtAGcAaTgTtGTATGG
			GAACcAtCaGAGAAgctTCGTTGGG
			GAAaGAtaTGAGAgTTATCtaCAGG
			GAAaGAACTGcaAAgcAaCGTTTGG
sg11	4	2	ATAAATGAATtACGTgAaGAaATGG
			ATAAATGAATGACaTTAaaAcTTGG
sg12	2	1	GAAAGGgAAAGGGAAAgGGAAAGGG
sg13	5	4	CACATGATtAAaATATAtTatCCGG
			CACATaAgAAAGAaAgAGTGgAAGG
			tACAgGATcAAcAgATAGTGCCCGG
			gACATGccAAAGcTATAGaGCTTGG
sg14	6	9	TATATtAAcTaTCaCTtTcACTAGG
			aATAaAAAATGTgGtTagGACATGG
			TATATgAcggGTtGgTGTGACCTGG
			TATATAcAATGTCGaTagagCTAGG
			TcTATAAAAaGTttCTGTtAgAGGG
			TtTATAtgATaTCGtTGTGcCATGG
			TtTgTAAAgTGTCGtcGTGAaTTGG
			TATATcAAAgGTataTGTGtCTAGG
			TAcAaAcAcTGTCGCTtTGAaTAGG
sg15	5	3	AAGGGTTtcCAAaTtAgTGACACGG
			AAGGGTcAAaAACaAAgTGtCACGG
			AAGGGaaAACgACTcACaGACGTGG
sg16	5	2	TTCAAgTGTTcAaGtAAGTaGATGG
			aTCAAATtTTAATtGAgGgGGTAGG
sg17	5	2	aGCAgAAAcAAgGACAAGGaATTGG
			GGaAaAAAAAAAtcaAAGGCACTGG
sg18	6	12	cGGtgAATTCaACtAAAACGaGGGG
			GactAAATTCttCCAAAAtGGTAGG
			GaGAAAcTTCGACaAAAgaaGGCGG
			tGGAAAAgTCttCCAAAAaaGGAGG
			atGAAAATTCtAtCAAcAtGGGGGG
			GGGAAAATTgGAaaAAAgaGaTGGG
			atGAAAATTCccCtAAAAtGGTTGG
			GaGAAAAaTgGACCgAtAtGGATGG
			GcGAAgATTCtACCAgAtgGGGCGG
			GGtAAAATgCtACCAcAttGGGTGG
			tGGAAAtcaaGACCAAtACGGAGGG

			tGGAgAAgTCGACgAtAAtGGTAGG
sg19	5	2	GaGATGAGaGAAACGtcAtGTATGG
			GaGATGAGCGAAAtGgAtaGTGGGG
sg20	5	3	TTTGTGTGTGtttGTAtAgCAACGG
			TTTGTGTcTGAACGTcaAaCtGAGG
			TTTaTaTGTGAAtGgACAaCAATGG
sg21	5	1	TATcAAAcGCGTCAttaCGCTCTGG
sg22	5	2	tTCGTcACAgCAAaGGGAAGaCGGG
			aTCaTAACcACAAcGGaAAGGGAGG
sg23	5	3	AgAAGTAaTAccGAGAGCTaAAAGG
			ATAAGTAccAcTGAGAGgaGAATGG
			ATAtaTtGTAgTGAGAGtTGATGGG
sg24	2	1	CCCTGGAATATGAAGGAgGtGGAGG

Supplementary Table 3. Functions of mutated ORFs in T1

**Contigs**	**Mutation Sites***	**ORFs**	**Variance of codons**	**Variance of amino acids**	**Summary of function**
1	163157	*BEM1*	GAC->GTC	Asp > Val	bud emergence protein 1
1	700435	*SLH1*	GAA->AAA	Glu > Lys	antiviral helicase SLH1processing
1	704513	*HER2*	CAG->CTG	Gln > Leu	glutamyl-tRNA(Gln) amidotransferase subunit A
1	768625	*SPC97*	ATT->AAT	Ile > Asn	spindle pole body component alp4
1	895916	*ERF4*	CAA->CAT	Gln > His	ras modification protein ERF4
1	1224656	*PYC2*	CAC->CGC	His > Arg	pyruvate carboxylase 2
1	1515155	*TFC4*	ATG->ATA	Met > Ile	transcription factor TFIIIC subunit
1	1539874	*TOF1*	GAA->TAA	Glu > *STOP*	opoisomerase 1-associated factor 1
2	85987	*VPS10*	AAA->ACA	Lys > Thr	vacuolar protein sorting/targeting protein PEP1
2	159940	*FES1*	ACG->ACT	*Synonymous*	hsp70 nucleotide exchange factor FES1
2	353985	*SIR4*	AGA->AAA	Arg > Lys	SIR4-interacting protein SIF2
2	1270758	*FIG4*	ATC->ATT	*Synonymous*	polyphosphoinositide phosphatase
2	844539	*USE1*	GCC->GCA	*Synonymous*	protein transport protein USE1
2	455693	*ESBP6*	CTG->CTA	*Synonymous*	uncharacterized transporter ESBP6
3	699660	*CDA2*	TGT->AGT	Cys > Ser	chitin deacetylase 2
3	859342	*RTS3*	AAC->AAA	Asn > Lys	Putative component of the protein

					phosphatase type 2A complex
3	1064780	*YNR021W*	+C	*Frameshift*	Affirmed Modeled Interactor 1
3	1272313	*PDE2*	TTC->TCC	Phe > Ser	cAMP phosphodiesterase
4	389170	*SEA3*	CAT->CTT	His > Leu	Maintenance of Telomere Capping
4	409746	*MDN1*	TTA->TCA	Leu > Ser	midasin
4	536297	*RPB5*	CAT->CCT	His > Pro	DNA-directed RNA polymerases I
4	578795	*RPF1*	AAT->AAA	Asn > Lys	ribosome production factor 1
4	582541	*PDR5*	TCT->GCT	Ser > Ala	ATP-dependent permease PDR15
4	582644	*PDR5*	TAC->TAT	*Synonymous*	ATP-dependent permease PDR15
4	582655	*PDR5*	GTG->TTG	Val > Leu	ATP-dependent permease PDR15
4	883547	*VSH2*	AAT->AAA	Asn > Lys	viable in a HAL3 SIT4 background protein 2
4	914268	*KRI1*	TTC->TCC	Phe > Ser	KRR1-Interacting protein
4	1065834	*IDP1*	GAA->AAA	Glu > Lys	isocitrate dehydrogenase [NADP]
4	1167172	*DUS1*	TTA->ATA	Leu > Ile	tRNA-dihydrouridine synthase 1
5	244881	*DRS2*	GTT->ATT	Val > Ile	probable phospholipid-transporting ATPase DRS2
5	244882	*DRS2*	GTT->GTC	*Synonymous*	probable phospholipid-transporting ATPase DRS2
6	1063497	*MTC6*	-G	*Frameshift*	Maintenance of Telomere

					Capping
7	260046	*APL4*	GCC->GCA	*Synonymous*	AP-1 complex subunit gamma-1
7	405705	*TWF1*	ATT->GTT	Ile > Val	twinfilin-1
7	510234	*HRD3*	GCC->ACC	Ala > Thr	ERAD-associated E3 ubiquitin-protein ligase component HRD3
7	876065	*ICL2*	TGG->TTG	Trp > Leu	mitochondrial 2-methylisocitrate lyase
8	192985	*CMS1*	GAT->GCT	Asp > Ala	protein CMS1
8	197329	*SSK1*	GAT->GCT	Asp > Ala	Suppressor of Sensor Kinase
8	206743	*YOR022C*	ACC->ACA	*Synonymous*	DDHD Domain-containing Lipase
8	566726	*TRM11*	AAG->AGG	Lys > Arg	multifunctional methyltransferase subunit TRM112

- Mutations in T1 were identified previously[1].

Supplementary Table 4. Plasmids used in this study.

Vector	Essential Features	Description	Sources
SlugCas9-HF	SlugCas9-HF, Sa gRNA scaffold	Expresses highly specific SlugCas9, and cloning backbone for sgRNA	[2]
pFA6a-13Myc-kanMX6	*kanMX*	Plasmid with a *kanMX* marker for adding a C-terminal 13xMyc tag.	[3]
pRS425-Cas9-2xSapI	2u origin, SpCas9, *LEU2, SNR52*promoter-(2xSapI site)-gRNA-*SUP4*	CRISPR vector of SpCas9 for gene editing in *S. cerevisiae*.	Bruce Futcher, Stony Brook University
LHZ531	*ARS1 /CEN5*, Cas9, *URA3*	CRIPSR vector of SpCas9 for gene editing in *K. Marxianus*	[4]
LHZ1493	*ARS1 /C*EN5, SlugCas9-HF, *URA3*	CRIPSR vector of SlugCas9-HF for gene editing in *K. Marxianus*	
LHZ1494	*ARS1/CEN5*, tRNAgly-sg1, SNR52-sg4, tRNAgly-sg7	Triple sgRNAs expression plasmids targeting sg1, sg4 and sg7.	
LHZ1495	*ARS1/CEN5*, SlugCas9-HF, *URA3*, sgADE2-1	CRISPR vector of SlugCas9-HF targeting the *ADE2* site 1.	
LHZ1496	*ARS1/CEN5*, SlugCas9-HF, *URA3*, sgADE2-2	CRISPR vector of SlugCas9-HF targeting the *ADE2* site 2.	
LHZ1497	*ARS1/CEN5*, SlugCas9-HF, *URA3*, sgADE2-3	CRISPR vector of SlugCas9-HF targeting the *ADE2* site 3.	
LHZ1498	*ARS1/CEN5*, SlugCas9-HF, *URA3*, sgADE2-4	CRISPR vector of SlugCas9-HF targeting the *ADE2* site 4.	
LHZ1499	*ARS1/CEN5*, SlugCas9-HF, *URA3*, sgADE2-5	CRISPR vector of SlugCas9-HF targeting the *ADE2* site 5.	
LHZ1500	*ARS1/CEN5*, SlugCas9-HF, *URA3*, sgADE2-6	CRISPR vector of SlugCas9-HF targeting the *ADE2* site 6.	
LHZ1501	*ARS1/CEN5*, SlugCas9-HF, *URA3*,	CRISPR vector of SlugCas9-HF targeting the *ADE2* site 7.	

	sgADE2-7		
LHZ1502	*ARS1/CEN5*, SlugCas9-HF, *URA3*, sgADE2-8	CRISPR vector of SlugCas9-HF targeting the *ADE2* site 8.	
LHZ1503	*ARS1/CEN5*, SlugCas9-HF, *URA3*, sg1RNA	CRISPR vector of SlugCas9-HF targeting the sg1 locus	
LHZ1504	*ARS1/CEN5*, SlugCas9-HF, *URA3*, sg2RNA	CRISPR vector of SlugCas9-HF targeting the sg2 locus	
LHZ1505	*ARS1/CEN5*, SlugCas9-HF, *URA3*, sg3RNA	CRISPR vector of SlugCas9-HF targeting the sg3 locus	
LHZ1506	*ARS1/CEN5*, SlugCas9-HF, *URA3*, sg4RNA	CRISPR vector of SlugCas9-HF targeting the sg4 locus	
LHZ1507	*ARS1/CEN5*, SlugCas9-HF, *URA3*, sg5RNA	CRISPR vector of SlugCas9-HF targeting the sg5 locus	
LHZ1508	*ARS1/CEN5*, SlugCas9-HF, *URA3*, sg6RNA	CRISPR vector of SlugCas9-HF targeting the sg6 locus	
LHZ1509	*ARS1/CEN5*, SlugCas9-HF, *URA3*, sg7RNA	CRISPR vector of SlugCas9-HF targeting the sg7 locus	
LHZ1510	*ARS1/CEN5*, SlugCas9-HF, *URA3*, sg8RNA	CRISPR vector of SlugCas9-HF targeting the sg8 locus	
LHZ1511	*ARS1/CEN5*, SlugCas9-HF, *URA3*, sg9RNA	CRISPR vector of SlugCas9-HF targeting the sg9 locus	
LHZ1512	*ARS1/CEN5*, SlugCas9-HF, *URA3*, sg10RNA	CRISPR vector of SlugCas9-HF targeting the sg10 locus	
LHZ1513	*ARS1/CEN5*, SlugCas9-HF, *URA3*, sg11RNA	CRISPR vector of SlugCas9-HF targeting the sg11 locus	
LHZ1514	*ARS1/CEN5*, SlugCas9-HF, *URA3*, sg12RNA	CRISPR vector of SlugCas9-HF targeting the sg12 locus	
LHZ1515	*ARS1/CEN5*, SlugCas9-HF, *URA3*, sg13RNA	CRISPR vector of SlugCas9-HF targeting the sg13 locus	

LHZ1516	*ARS1/CEN5*, SlugCas9-HF, *URA3*, sg14RNA	CRISPR vector of SlugCas9-HF targeting the sg14 locus	
LHZ1517	*ARS1/CEN5*, SlugCas9-HF, *URA3*, sg15RNA	CRISPR vector of SlugCas9-HF targeting the sg15 locus	
LHZ1518	*ARS1/CEN5*, SlugCas9-HF, *URA3*, sg16RNA	CRISPR vector of SlugCas9-HF targeting the sg16 locus	
LHZ1519	*ARS1/CEN5*, SlugCas9-HF, *URA3*, sg17RNA	CRISPR vector of SlugCas9-HF targeting the sg17 locus	
LHZ1520	*ARS1/CEN5*, SlugCas9-HF, *URA3*, sg18RNA	CRISPR vector of SlugCas9-HF targeting the sg18 locus	
LHZ1521	*ARS1/CEN5*, SlugCas9-HF, *URA3*, sg19RNA	CRISPR vector of SlugCas9-HF targeting the sg19 locus	
LHZ1522	*ARS1/CEN5*, SlugCas9-HF, *URA3*, sg20RNA	CRISPR vector of SlugCas9-HF targeting the sg20 locus	
LHZ1523	*ARS1/CEN5*, SlugCas9-HF, *URA3*, sg21RNA	CRISPR vector of SlugCas9-HF targeting the sg21 locus	
LHZ1524	*ARS1/CEN5*, SlugCas9-HF, *URA3*, sg22RNA	CRISPR vector of SlugCas9-HF targeting the sg22 locus	
LHZ1525	*ARS1/CEN5*, SlugCas9-HF, *URA3*, sg23RNA	CRISPR vector of SlugCas9-HF targeting the sg23 locus	
LHZ1526	*ARS1/CEN5*, SlugCas9-HF, *URA3*, sg24RNA	CRISPR vector of SlugCas9-HF targeting the sg24 locus	
LHZ1527	*ARS1/CEN5*, SlugCas9-HF, *URA3*, sg303M1	CRISPR vector of SlugCas9-HF targeting the off-target site between *ISA1* and *RIM4* gene.	
LHZ1528	*ARS1/CEN5*, SlugCas9-HF, *URA3*, sg303M2	CRISPR vector of SlugCas9-HF targeting the off-target site between *ISA1* and *RIM4* gene.	
LHZ1529	*ARS1/CEN5*, SlugCas9-HF, *URA3*, sg303M3	CRISPR vector of SlugCas9-HF targeting the off-target site between *ISA1* and *RIM4* gene.	

LHZ1530	*ARS1/CEN5*, SlugCas9-HF, *URA3*, sg303M4	CRISPR vector of SlugCas9-HF targeting the off-target site between *ISA1* and *RIM4* gene.	
LHZ1531	*ARS1/CEN5*, SlugCas9-HF, *URA3*, sg303M5	CRISPR vector of SlugCas9-HF targeting the off-target site between *ISA1* and *RIM4* gene.	
LHZ1532	*ARS1/CEN5*, SlugCas9-HF, *URA3*, sg303M6	CRISPR vector of SlugCas9-HF targeting the off-target site between *ISA1* and *RIM4* gene.	
LHZ1533	*ARS1/CEN5*, SlugCas9-HF, *URA3*, sg303M7	CRISPR vector of SlugCas9-HF targeting the off-target site between *ISA1* and *RIM4* gene.	
LHZ1534	*ARS1/CEN5*, SpCas9, *URA3*, sg303M1	CRISPR vector of SpCas9 targeting the off-target site between *ISA1* and *RIM4* gene.	
LHZ1535	*ARS1/CEN5*, SpCas9, *URA3*, sg303M2	CRISPR vector of SpCas9 targeting the off-target site between *ISA1* and *RIM4* gene.	
LHZ1536	*ARS1/CEN5*, SpCas9, *URA3*, sg303M3	CRISPR vector of SpCas9 targeting the off-target site between *ISA1* and *RIM4* gene.	
LHZ1537	*ARS1/CEN5*, SpCas9, *URA3*, sg303M4	CRISPR vector of SpCas9 targeting the off-target site between *ISA1* and *RIM4* gene.	
LHZ1538	*ARS1/CEN5*, SpCas9, *URA3*, sg303M5	CRISPR vector of SpCas9 targeting the off-target site between *ISA1* and *RIM4* gene.	
LHZ1539	*ARS1/CEN5*, SpCas9, *URA3*, sg303M6	CRISPR vector of SpCas9 targeting the off-target site between *ISA1* and *RIM4* gene.	
LHZ1540	*ARS1/CEN5*, SpCas9, *URA3*, sg303M7	CRISPR vector of SpCas9 targeting the off-target site between *ISA1* and *RIM4* gene.	
LHZ1541	*ARS1/CEN5*, SlugCas9-HF, *URA3*, sgADE2M1	CRISPR vector of SlugCas9-HF targeting the off-target site in *ADE2* gene.	
LHZ1542	*ARS1/CEN5*, SlugCas9-HF, *URA3*, sgADE2M2	CRISPR vector of SlugCas9-HF targeting the off-target site in *ADE2* gene.	
LHZ1543	*ARS1/CEN5*, SlugCas9-HF, *URA3*, sgADE2M3	CRISPR vector of SlugCas9-HF targeting the off-target site in *ADE2* gene.	

LHZ1544	*ARS1/CEN5*, SlugCas9-HF, *URA3*, sgADE2M4	CRISPR vector of SlugCas9-HF targeting the off-target site in *ADE2* gene.	
LHZ1545	*ARS1/CEN5*, SlugCas9-HF, *URA3*, sgADE2M5	CRISPR vector of SlugCas9-HF targeting the off-target site in *ADE2* gene.	
LHZ1546	*ARS1/CEN5*, SlugCas9-HF, *URA3*, sgADE2M6	CRISPR vector of SlugCas9-HF targeting the off-target site in *ADE2* gene.	
LHZ1547	*ARS1/CEN5*, SlugCas9-HF, *URA3*, sgADE2M7	CRISPR vector of SlugCas9-HF targeting the off-target site in *ADE2* gene.	
LHZ1548	*ARS1/CEN5*, SpCas9, *URA3*, sgADE2M1	CRISPR vector of SpCas9 targeting the off-target site in *ADE2* gene.	
LHZ1549	*ARS1/CEN5*, SpCas9, *URA3*, sgADE2M2	CRISPR vector of SpCas9 targeting the off-target site in *ADE2* gene.	
LHZ1550	*ARS1/CEN5*, SpCas9, *URA3*, sgADE2M3	CRISPR vector of SpCas9 targeting the off-target site in *ADE2* gene.	
LHZ1551	*ARS1/CEN5*, SpCas9, *URA3*, sgADE2M4	CRISPR vector of SpCas9 targeting the off-target site in *ADE2* gene.	
LHZ1552	*ARS1/CEN5*, SpCas9, *URA3*, sgADE2M5	CRISPR vector of SpCas9 targeting the off-target site in *ADE2* gene.	
LHZ1553	*ARS1/CEN5*, SpCas9, *URA3*, sgADE2M6	CRISPR vector of SpCas9 targeting the off-target site in *ADE2* gene.	
LHZ1554	*ARS1/CEN5*, SpCas9, *URA3*, sgADE2M7	CRISPR vector of SpCas9 targeting the off-target site in *ADE2* gene.	
LHZ1555	AmpR	Donor helper plasmids containing upstream and downstream homology arms targeting the sg1 locus	
LHZ1556	AmpR	Donor helper plasmids containing upstream and downstream homology arms targeting the sg4 locus	
LHZ1557	AmpR	Donor helper plasmids containing upstream and downstream homology arms targeting the sg7	

		locus	
LHZ1558	AmpR	Donor helper plasmids containing upstream and downstream homology arms targeting the sg8 locus	
LHZ1559	AmpR	Donor helper plasmids containing upstream and downstream homology arms targeting the sg19 locus	
LHZ1560	AmpR	Donor helper plasmids containing upstream and downstream homology arms targeting the sg23 locus	
LHZ1561	*ARS1/CEN5*, *URA3*	Empty CRIPSR vector without SlugCas9-HF protein based on LHZ1493.	

Supplementary Table 5. Primers used in this study.

SyM-A-F	CTGGAAGAACAGTGGTATTCTAGCCGGgcTC	Construction of LHZ1493
SyM-A-R	GACCAGCCGGATGCATTTCAGG	
New-1-F	CAAGAAGAGCCATGGCTCTTCAGGTTAATTAAGCACTGGCCGTCGTTTTAC	
CPSlugCas9-ARS1-R	GGCCATTTTTCCCGTTCTAGAAAACT	
SlugCas9-ARS1-F	CTAAGTTTTCTAGAACGGGAAAAATGGCCCCAAAGAAGAAGCGGAAG	
SlugCas9-ARS1-R	CTAATTACATGACTCGAGAAGAGACTTTTTCTTTTTTGCCTGGCCGGC	
CPSlugCas9-ARS1-F	TCTCTTCTCGAGTCATGTAATTAGT	
New-3-R	CTGAAGAGCCATGGCTCTTCTTG	
CPWhole-F	GTTTTAGTACTCTGGAAACAGAATC	
CPWhole-R	CGACGGCCAGTGCTTAATTAACTAATACGACTCACTATAGGGCGAAT	
Whole-F	GTTAATTAAGCACTGGCCGTCGTTTTAC	
Whole-R	CTGTTTCCAGAGTACTAAAACCTGAAGAGCCATGGCTCTTCTTG	
emptyCas9-F	CCGCTTATACTCCAACTTGGTC	Construction of LHZ1561
emptyCas9-R	CGCGTTAACGAGCTCCTTTC	
slug-ADE2-1gF	TCAACAATATAAACCTTGTTAATC	Construction of LHZ1495~1502
slug-ADE2-1gR	AACGATTAACAAGGTTTATATTGT	
slug-ADE2-2gF	TCAGATTTGGACCTGCTTAAGAGT	
slug-ADE2-2gR	AACACTCTTAAGCAGGTCCAAATC	
slug-ADE2-3gF	TCATTTCCTCTGCCATCATAAGCA	
slug-ADE2-3gR	AACTGCTTATGATGGCAGAGGAAA	
slug-ADE2-4gF	TCACACTGAGTCTGGAACTCTAGC	
slug-ADE2-	AACGCTAGAGTTCCAGACTCAGTG	

4gR		
slug-ADE2-5gF	TCATACACCGAAAATACCAGCACC	
slug-ADE2-5gR	AACGGTGCTGGTATTTTCGGTGTA	
slug-ADE2-6gF	TCAATGGTATAATGGCCGGAATTA	
slug-ADE2-6gR	AACTAATTCCGGCCATTATACCAT	
slug-ADE2-7gF	TCAAACCATTAGTTAGCGTTATTA	
slug-ADE2-7gR	AACTAATAACGCTAACTAATGGTT	
slug-ADE2-8gF	TCAGACATTCTGTGAGGAGTTCTA	
slug-ADE2-8gR	AACTAGAACTCCTCACAGAATGTC	
mono303-slug-M0F	TCAAGTTTCCGAGGGAGGTTGCCG	Construction of LHZ1527~1540
mono303-slug-M1F	TCAACTTTCCGAGGGAGGTTGCCG	
mono303-slug-M2F	TCAAGTTACCGAGGGAGGTTGCCG	
mono303-slug-M3F	TCAAGTTTCCCAGGGAGGTTGCCG	
mono303-slug-M4F	TCAAGTTTCCGAGCGAGGTTGCCG	
mono303-slug-M5F	TCAAGTTTCCGAGGGACGTTGCCG	
mono303-slug-M6F	TCAAGTTTCCGAGGGAGGTAGCCG	
mono303-slug-M7F	TCAAGTTTCCGAGGGAGGTTGCGG	
mono303-slug-M0R	AACCGGCAACCTCCCTCGGAAACT	
mono303-slug-M1R	AACCGGCAACCTCCCTCGGAAAGT	
mono303-slug-M2R	AACCGGCAACCTCCCTCGGTAACT	
mono303-slug-M3R	AACCGGCAACCTCCCTGGGAAACT	
mono303-slug-M4R	AACCGGCAACCTCGCTCGGAAACT	

mono303-slug-M5R	AACCGGCAACGTCCCTCGGAAACT	
mono303-slug-M6R	AACCGGCTACCTCCCTCGGAAACT	
mono303-slug-M7R	AACCCGCAACCTCCCTCGGAAACT	
mono303-sp-M0F	TCAGTTTCCGAGGGAGGTTGCCG	
mono303-sp-M1F	TCACTTTCCGAGGGAGGTTGCCG	
mono303-sp-M2F	TCAGTTACCGAGGGAGGTTGCCG	
mono303-sp-M3F	TCAGTTTCCCAGGGAGGTTGCCG	
mono303-sp-M4F	TCAGTTTCCGAGCGAGGTTGCCG	
mono303-sp-M5F	TCAGTTTCCGAGGGACGTTGCCG	
mono303-sp-M6F	TCAGTTTCCGAGGGAGGTAGCCG	
mono303-sp-M7F	TCAGTTTCCGAGGGAGGTTGCGG	
mono303-sp-M0R	AACCGGCAACCTCCCTCGGAAAC	
mono303-sp-M1R	AACCGGCAACCTCCCTCGGAAAG	
mono303-sp-M2R	AACCGGCAACCTCCCTCGGTAAC	
mono303-sp-M3R	AACCGGCAACCTCCCTGGGAAAC	
mono303-sp-M4R	AACCGGCAACCTCGCTCGGAAAC	
mono303-sp-M5R	AACCGGCAACGTCCCTCGGAAAC	
mono303-sp-M6R	AACCGGCTACCTCCCTCGGAAAC	
mono303-sp-M7R	AACCCGCAACCTCCCTCGGAAAC	
mono4ADE2-slug-M1F	TCATTCACCGAAAATACCAGCACC	Construction of LHZ1541~1554
mono4ADE2-slug-M2F	TCATACAGCGAAAATACCAGCACC	
mono4ADE2-slug-M3F	TCATACACCGTAAATACCAGCACC	

mono4ADE 2-slug-M4F	TCATACACCGAAATTACCAGCACC	
mono4ADE 2-slug-M5F	TCATACACCGAAAATAGCAGCACC	
mono4ADE 2-slug-M6F	TCATACACCGAAAATACCACCACC	
mono4ADE 2-slug-M7F	TCATACACCGAAAATACCAGCAGC	
mono4ADE 2-slug-M1R	AACGGTGCTGGTATTTTCGGTGAA	
mono4ADE 2-slug-M2R	AACGGTGCTGGTATTTTCGCTGTA	
mono4ADE 2-slug-M3R	AACGGTGCTGGTATTTACGGTGTA	
mono4ADE 2-slug-M4R	AACGGTGCTGGTAATTTCGGTGTA	
mono4ADE 2-slug-M5R	AACGGTGCTGCTATTTTCGGTGTA	
mono4ADE 2-slug-M6R	AACGGTGGTGGTATTTTCGGTGTA	
mono4ADE 2-slug-M7R	AACGCTGCTGGTATTTTCGGTGTA	
mono4ADE 2-sp-M1F	TCATCACCGAAAATACCAGCACC	
mono4ADE 2-sp-M2F	TCAACAGCGAAAATACCAGCACC	
mono4ADE 2-sp-M3F	TCAACACCGTAAATACCAGCACC	
mono4ADE 2-sp-M4F	TCAACACCGAAATTACCAGCACC	
mono4ADE 2-sp-M5F	TCAACACCGAAAATAGCAGCACC	
mono4ADE 2-sp-M6F	TCAACACCGAAAATACCACCACC	
mono4ADE 2-sp-M7F	TCAACACCGAAAATACCAGCAGC	
mono4ADE 2-sp-M1R	AACGGTGCTGGTATTTTCGGTGA	
mono4ADE 2-sp-M2R	AACGGTGCTGGTATTTTCGCTGT	
mono4ADE 2-sp-M3R	AACGGTGCTGGTATTTACGGTGT	
mono4ADE 2-sp-M4R	AACGGTGCTGGTAATTTCGGTGT	

mono4ADE 2-sp-M5R	AACGGTGCTGCTATTTTCGGTGT	
mono4ADE 2-sp-M6R	AACGGTGGTGGTATTTTCGGTGT	
mono4ADE 2-sp-M7R	AACGCTGCTGGTATTTTCGGTGT	
NGS-sg1-F	ACACTCTTTCCCTACACGACGCTCTTCCGATCT NNNNTGGCATATACACAAGCTTACTCC	Primers to amplify each intergenic locus for deep sequencing of step I.
NGS-sg3-F	ACACTCTTTCCCTACACGACGCTCTTCCGATCT NNNNTCCTGGTAACTTGTAACTTGTAACT	
NGS-sg4-F	ACACTCTTTCCCTACACGACGCTCTTCCGATCT NNNNACTGCAAAGCATGCGAGAGG	
NGS-sg5-F	ACACTCTTTCCCTACACGACGCTCTTCCGATCT NNNNAGAAAAACAGTAGTGGAAGGAAGGA	
NGS-sg6-F	ACACTCTTTCCCTACACGACGCTCTTCCGATCT NNNNCCTGCGTTTTCCTGCAAAGT	
NGS-sg7&8 -F	ACACTCTTTCCCTACACGACGCTCTTCCGATCT NNNNGGCAAAATTAGCCGCAGAAGT	
NGS-sg9&1 0-F	ACACTCTTTCCCTACACGACGCTCTTCCGATCT NNNNACAGGATGGGGAGATCTGATG	
NGS-sg11- F	ACACTCTTTCCCTACACGACGCTCTTCCGATCT NNNNCTTTCACCGCTGCCCATTG	
NGS-sg12& 13-F	ACACTCTTTCCCTACACGACGCTCTTCCGATCT NNNNGTTTCGAGAATTAGTTTCAGATGCG	
NGS-sg14- F	ACACTCTTTCCCTACACGACGCTCTTCCGATCT NNNNGAGTTCGGAATTCCAGGCCA	
NGS-sg15- F	ACACTCTTTCCCTACACGACGCTCTTCCGATCT NNNNTGGACGTCAGTTGATGTTCTT	
NGS-sg16& 17-F	ACACTCTTTCCCTACACGACGCTCTTCCGATCT NNNNCATCAACTGACGTCCATAACTGAT	
NGS-sg18& 19-F	ACACTCTTTCCCTACACGACGCTCTTCCGATCT NNNNGAGTGAAGAAAGTCCCAAGTGT	
NGS-sg20- F	ACACTCTTTCCCTACACGACGCTCTTCCGATCT NNNNAGGGCGGGTTTCTTTCCTTC	

NGS-sg21-F	ACACTCTTTCCCTACACGACGCTCTTCCGATCT NNNNCAACTTGCGCTCACATTGCTT	
NGS-sg22-F	ACACTCTTTCCCTACACGACGCTCTTCCGATCT NNNNGCATATATAAAAAGCGTCAGAGCG	
NGS-sg23-F	ACACTCTTTCCCTACACGACGCTCTTCCGATCT NNNNAATTTGGGGCAGCTTAGTATCG	
NGS-sg24-F	ACACTCTTTCCCTACACGACGCTCTTCCGATCT NNNNCCAGAAGGGGCGAAGTCTAC	
NGS-sg1-R	ACTGGAGTTCAGACGTGTGCTCTTCCGATCTNN NNTTCCCTATTGCCCCTAGCTT	
NGS-sg3-R	ACTGGAGTTCAGACGTGTGCTCTTCCGATCTNN NNCTGCTTCCATCAACGCCAAG	
NGS-sg4-R	ACTGGAGTTCAGACGTGTGCTCTTCCGATCTNN NNTGACGATCCCCGTGTAAATAGAG	
NGS-sg5R	ACTGGAGTTCAGACGTGTGCTCTTCCGATCTNN NNAGTGCCAGGACAGATGATTGA	
NGS-sg6-R	ACTGGAGTTCAGACGTGTGCTCTTCCGATCTNN NNGAGTGACAGACACTTCAAACTGA	
NGS-sg7&8-R	ACTGGAGTTCAGACGTGTGCTCTTCCGATCTNN NNAAGGCAGCAACGAATCACAA	
NGS-sg9&10-R	ACTGGAGTTCAGACGTGTGCTCTTCCGATCTNN NNATCAAGCAAGACGCAAGCATTA	
NGS-sg11-R	ACTGGAGTTCAGACGTGTGCTCTTCCGATCTNN NNTTCGATCCTGAGTGCGAGC	
NGS-sg12&13-R	ACTGGAGTTCAGACGTGTGCTCTTCCGATCTNN NNACCCGTTTAGCTTTGCCTGG	
NGS-sg14-R	ACTGGAGTTCAGACGTGTGCTCTTCCGATCTNN NNTCAGTCCTTCTCTTCTTACGATATG	
NGS-sg15-R	ACTGGAGTTCAGACGTGTGCTCTTCCGATCTNN NNGTACTGCGTTGGCAGGACT	
NGS-sg16&17-R	ACTGGAGTTCAGACGTGTGCTCTTCCGATCTNN NNAAGACAAGGTTGAAGGGGGATT	

NGS-sg18&19-R	ACTGGAGTTCAGACGTGTGCTCTTCCGATCTNNNNATGCGGGATTTTGTGTGTGAAC	
NGS-sg20-R	ACTGGAGTTCAGACGTGTGCTCTTCCGATCTNNNNCCAAAACGGAAGGGGGCATA	
NGS-sg21-R	ACTGGAGTTCAGACGTGTGCTCTTCCGATCTNNNNCTTCCTTCCCCTTGTTGTTACG	
NGS-sg22-R	ACTGGAGTTCAGACGTGTGCTCTTCCGATCTNNNNAATGAATACAAAACACGAACCGT	
NGS-sg23-R	ACTGGAGTTCAGACGTGTGCTCTTCCGATCTNNNNGGTCATGGCGAACCTAATCAA	
NGS-sg24-R	ACTGGAGTTCAGACGTGTGCTCTTCCGATCTNNNNGATTTGGATTCCAGTACAGTAAAGT	
NGS-ADE2-F	ACACTCTTTCCCTACACGACGCTCTTCCGATCTNNNNTGGGCTTCGAATTGAGAGGT	
NGS-ADE2-R	ACTGGAGTTCAGACGTGTGCTCTTCCGATCTNNNNCGACGGTAGAGACCATCCAC	
P5-index1-F	AATGATACGGCGACCACCGAGATCTACACTGAACCTTACACTCTTTCCCTACACGAC	Primers to amplify the intergenic locus for deep sequencing of step II
P5-index2-F	AATGATACGGCGACCACCGAGATCTACAC TGCTAAGT ACACTCTTTCCCTACACGAC	
P5-index3-F	AATGATACGGCGACCACCGAGATCTACAC TAAGACAC ACACTCTTTCCCTACACGAC	
P5-index4-F	AATGATACGGCGACCACCGAGATCTACAC TGTTCTCT ACACTCTTTCCCTACACGAC	
P5-index5-F	AATGATACGGCGACCACCGAGATCTACAC CTAATCGA ACACTCTTTCCCTACACGAC	
P5-index6-F	AATGATACGGCGACCACCGAGATCTACAC CTAGAACA ACACTCTTTCCCTACACGAC	
P7-adapter 26-R	CAAGCAGAAGACGGCATACGAGAT TCTCCGGA GTGACTGGAGTTCAGACGTGTG	
P7-adapter 27-R	CAAGCAGAAGACGGCATACGAGAT AATGAGCG GTGACTGGAGTTCAGACGTGTG	

P7-adapter 28-R	CAAGCAGAAGACGGCATACGAGAT GGAATCTC GTGACTGGAGTTCAGACGTGTG	
P7-adapter 29-R	CAAGCAGAAGACGGCATACGAGAT TTCTGAAT GTGACTGGAGTTCAGACGTGTG	
P7-adapter 30-R	CAAGCAGAAGACGGCATACGAGAT ACGAATTC GTGACTGGAGTTCAGACGTGTG	
P7-adapter 31-R	CAAGCAGAAGACGGCATACGAGAT AGCTTCAG GTGACTGGAGTTCAGACGTGTG	
P7-adapter 32-R	CAAGCAGAAGACGGCATACGAGAT GCGCATTA GTGACTGGAGTTCAGACGTGTG	
P7-adapter 33-R	CAAGCAGAAGACGGCATACGAGAT CATAGCCG GTGACTGGAGTTCAGACGTGTG	
P7-adapter 34-R	CAAGCAGAAGACGGCATACGAGAT TTCGCGGA GTGACTGGAGTTCAGACGTGTG	
P7-adapter 35-R	CAAGCAGAAGACGGCATACGAGAT GCGCGAGA GTGACTGGAGTTCAGACGTGTG	
P7-adapter 36-R	CAAGCAGAAGACGGCATACGAGAT CTATCGCT GTGACTGGAGTTCAGACGTGTG	
P7-adapter 37-R	CAAGCAGAAGACGGCATACGAGAT GTCGTGAT GTGACTGGAGTTCAGACGTGTG	
P7-adapter 40-R	CAAGCAGAAGACGGCATACGAGAT CCGTTTGT GTGACTGGAGTTCAGACGTGTG	
HP-pUC19-F	CATCCGCTTACAGACAAGCTG	Construction of LHZ1555~1560
HP-PUC19-R	CTTCCTCGCTCACTGACTCG	
HD-GFP-F	AATCCAGCACGAATCCACTATACACC	
HD-GFP-R	TGCCGGTAGAGGTGTGGT	
pUC19-sg1u-F	CGAGTCAGTGAGCGAGGAAGGAAGCACTTGGACCTATTG	
pUC19-sg4u-F	CGAGTCAGTGAGCGAGGAAGATCATTGATCCTTTCAAGGTTG	
pUC19-sg7u-F	CGAGTCAGTGAGCGAGGAAGGTAATAGTAGTGAGTGACAGACAC	

pUC19-sg8u-F	CGAGTCAGTGAGCGAGGAAGACAGACACTTCAAACTGAAATCAGTCCAC	
pUC19-sg19u-F	CGAGTCAGTGAGCGAGGAAGCTAATCAATAAGCGAAATACTCCCAC	
pUC19-sg23u-F	CGAGTCAGTGAGCGAGGAAGCATTCAACGGTGGTGTCTAC	
HDsg1-up-R	TAGTGGATTCGTGCTGGATT AATTCCCCCAATTTACCT	
HDsg4-up-R	TAGTGGATTCGTGCTGGATT GGAAGGAAATATAATTTACTACTTTCAATC	
HDsg7-up-R	TAGTGGATTCGTGCTGGATT ATACCAAGGTAATTAATTTCCACTG	
HDsg8-up-R	TAGTGGATTCGTGCTGGATT AGGGCGGTTACCAGGGAAATG	
HDsg19-up-R	TAGTGGATTCGTGCTGGATT TAAGGGAAAACTGGGCAATC	
HDsg23-up-R	TAGTGGATTCGTGCTGGATT TGAGAGCTGATAGGGTTG	
HDsg1-down-F	TGACCACACCTCTACCGGCA ATAAGCGTATGGGCATAG	
HDsg4-down-F	TGACCACACCTCTACCGGCA TTCCACTACTGTTTTTCTATTGATG	
HDsg7-down-F	TGACCACACCTCTACCGGCA TTCCAATGCTGTGTACAACC	
HDsg8-down-F	TGACCACACCTCTACCGGCA CGGCAACCTCCCTCGGAAAC	
HDsg19-down-F	TGACCACACCTCTACCGGCA CGTTTCGTTTCGCTCATCC	
HDsg23-down-F	TGACCACACCTCTACCGGCA ATACTACTTATTACCGTTTGCTTG	
sg1d-pUC19-R	CAGCTTGTCTGTAAGCGGATGGACTCATGAGTTGGATCTC	
sg4d-pUC19-R	CAGCTTGTCTGTAAGCGGATGAGCGATAGATATAATGGAGTTGAAC	
sg7d-pUC19-R	CAGCTTGTCTGTAAGCGGATGGATTTCACTCCCTCCATTGTG	
sg8d-pUC19-R	CAGCTTGTCTGTAAGCGGATGAGCTTCTGCTTCTGCTCCTGTG	
sg19d-pUC19-R	CAGCTTGTCTGTAAGCGGATGGTTAGCTAGCTAGTCTTACGTG	
sg23d-pUC19-R	CAGCTTGTCTGTAAGCGGATGCCTGTGAAGGAATGTGTTG	

sg1u-F	GAAGCACTTGGACCTATTG	Primers to amplify donor DNA fragments from the donor plasmids
sg4u-F	ATCATTGATCCTTTCAAGGTTG	
sg7u-F	GTAATAGTAGTGAGTGACAGACAC	
sg8u-F	ACAGACACTTCAAACTGAAATCAGTCCAC	
sg19u-F	CTAATCAATAAGCGAAATACTCCCAC	
sg23u-F	CATTCAACGGTGGTGTCTAC	
sg1d-R	GACTCATGAGTTGGATCTC	
sg4d-R	AGCGATAGATATAATGGAGTTGAAC	
sg7d-R	GATTTCACTCCCTCCATTGTG	
sg8d-R	AGCTTCTGCTTCTGCTCCTGTG	
sg19d-R	GTTAGCTAGCTAGTCTTACGTG	
sg23d-R	CCTGTGAAGGAATGTGTTG	
veri-sg1-F	ACTCCGACTACATCAAGGTC	Primers to test whether GFP cassettes were integrated into the target sites.
veri-sg1-R	CTCTACGGCTTGGCTATCG	
veri-sg4-F	CATCAGAGTTGGTGGCTCTTC	
veri-sg4-R	TTGGGAAAAGAGGAAATGGTTG	
veri-sg7-F	GATATGGGTGCCAGACCATTG	
veri-sg7-R	GCTTCTGCTCCTGTGGACATAG	
veri-sg7-F	GATATGGGTGCCAGACCATTG	
veri-sg8-R	AGTCAACCAGGATTCATGAC	
veri-sg19-F	GAAGACAAGTGCCACGAATCC	
veri-sg19-R	GACGGTGAGATCATCTACAAGG	
veri-sg23-F	CACGAGTTCCCAACTGACTAC	
veri-sg23-R	GGCACTATTCAAGAAGGAAC	
veri-sg1-up F	GTTGTTCTAACTGAAACCGGTACC	Primers to test whether the four lengths of gene fragments were integrated into sg1 locus.
veri-Sfrag-upR	ATACGATGATGCAACAGGC	
veri-3kfrag-upR	CTACATTACGTGACGTTACCCTC	
veri-6kfrag-upR	GCATTCTTATTGCTTCTCCAATCTCG	
veri-Lfrag-upR	GTATGTGGGAGTGTGTTTGTCG	
sg1-tRNA-F	AATGAAAGGAGCTCGTCGACAATTTCTCTTCTACCACGAACTC	Construction of LHZ1494.
sg1-tRNA-R	GACAAGGCAAGCTAAACAGACCAGTGCTTAATTAACTAATACGAC	
sg7-tRNA-F	AATTTCTCTTCTACCACGAACTC	
sg7-tRNA-R	GATCCGCGGCCGCATAGGCCCTATAGGGCGAATTGGGTAC	

sg4-SNR52-F	GTGAGTCGTATTAGTTAATTAACGCTTCTTTGAAAAGATAATGTATG	
sg4-SNR52-R	CAAGCTAAACAGACCAGTGCCTATAGGGCGAATTGGGTAC	
sg1-upR-Frag1	ACTTCCGGAATCGGGAGCGCAATTCCCCCAATTTACCTTTATTTAATTC	Construction of the 16 heme pathway donor plasmids.
sg1-downF-Frag1	CTCCCGCCGTGCACAGGGTGATAAGCGTATGGGCATAG	
sg1-upR-Frag2	ACAAGGCCATTCATTATTCAAATTCCCCCAATTTACCTTTATTTAATTC	
sg1-downF-Frag2	CAATTTCCCAAAAAAACAAGATAAGCGTATGGGCATAG	
sg4-upR-Frag1	ACTTCCGGAATCGGGAGCGCGGAAGGAAATATAATTTACTACTTTCAATC	
sg4-downF-Frag1	CTCCCGCCGTGCACAGGGTGATAAGCGTATGGGCATAGTTCCACTACTGTTTTTCTATTGATG	
sg4-upR-Frag2	ACAAGGCCATTCATTATTCACGGAAGGAAATATAATTTACTACTTTCAATC	
sg4-downF-Frag2	CAATTTCCCAAAAAAACAAGTTCCACTACTGTTTTTCTATTGATG	
sg7-upR-Frag1	ACTTCCGGAATCGGGAGCGCAATTCCCCCAATTTACCTTTATTTAATTCATACCAAGGTAATTAATTTCCACTG	
sg7-downF-Frag1	CTCCCGCCGTGCACAGGGTGATAAGCGTATGGGCATAGTTCCAATGCTGTGTACAACC	
sg7-upR-Frag2	ACAAGGCCATTCATTATTCAATACCAAGGTAATTAATTTCCACTG	
sg7-downF-Frag2	CAATTTCCCAAAAAAACAAGTTCCAATGCTGTGTACAACC	
sg19-upR-Frag1	ACTTCCGGAATCGGGAGCGCAATTCCCCCAATTTACCTTTATTTAATTCTAAGGGAAAACTGGGCAATC	
sg19-downF-Frag1	CTCCCGCCGTGCACAGGGTGATAAGCGTATGGGCATAGCGTTTCGTTTCGCTCATCC	
sg19-upR-Frag2	ACAAGGCCATTCATTATTCATAAGGGAAAACTGGGCAATC	
sg19-downF-Frag2	CAATTTCCCAAAAAAACAAGCGTTTCGTTTCGCTCATCC	
sg1-upR-Frag3	GTTTTTTTGGGAAATTGAAAATAATTCCCCCAATTTACCTTTATTTAATTC	

sg1-downF-Frag3	CCAACATTCTAGGATCGGTATAAGCGTATGGGCATAG	
sg1-upR-Frag4	ATTACGTACATTCACCTGGCAATTCCCCCAATTTACCTTTATTTAATTC	
sg1-downF-Frag4	GTAGTACTACTACCATTACATAAGCGTATGGGCATAG	
sg4-upR-Frag3	GTTTTTTTGGGAAATTGAAAATGGAAGGAAATATAATTTACTACTTTCAATC	
sg4-downF-Frag3	CCAACATTCTAGGATCGGTTTCCACTACTGTTTTTCTATTGATG	
sg4-upR-Frag4	ATTACGTACATTCACCTGGCGGAAGGAAATATAATTTACTACTTTCAATC	
sg4-downF-Frag4	AGTAGTACTACTACCATTACTTCCACTACTGTTTTTCTATTGATG	
sg7-upR-Frag3	GTTTTTTTGGGAAATTGAAAATATACCAAGGTAATTAATTTCCACTG	
sg7-downF-Frag3	CCAACATTCTAGGATCGGTTTCCAATGCTGTGTACAACC	
sg7-upR-Frag4	ATTACGTACATTCACCTGGCATACCAAGGTAATTAATTTCCACTG	
sg7-downF-Frag4	AGTAGTACTACTACCATTACTTCCAATGCTGTGTACAACC	
sg19-upR-Frag3	GTTTTTTTGGGAAATTGAAAATTAAGGGAAAACTGGGCAATC	
sg19-downF-Frag3	GACCAACATTCTAGGATCGGTCGTTTCGTTTCGCTCATCC	
sg19-upR-Frag4	ATTACGTACATTCACCTGGCTAAGGGAAAACTGGGCAATC	
sg19-downF-Frag4	AGTAGTACTACTACCATTACCGTTTCGTTTCGCTCATCC	
Frag1-F	GCGCTCCCGATTCCGGAAGT	Primers to amplify the 16 heme pathway donors from the plasmids.
Frag1-R	CACCCTGTGCACGGCGGG	
Frag2-F	TGAATAATGAATGGCCTTGTATTCG	
Frag2-R	CTTGTTTTTTTGGGAAATTGAAAATG	
Frag3-F	ATTTTCAATTTCCCAAAAAAACAAGAAGCC	
Frag3-R	ACCGATCCTAGAATGTTGGTCAG	
Frag4-F	GCCAGGTGAATGTACGTAATGG	
Frag4-R	GTAATGGTAGTAGTACTACTGCTACGAG	
P1-R	GTATGTGGGAGTGTGTTTGTCG	Primers to

P2-R	CATACCATGCCATGGCCATATTC	test whether heme genes were integrated into the target sites.
P3-R	CCATGATATTGGTGATGCTCGC	
P4-R	GCGACTAGGTTAGCCCTATTG	

Supplementary References

1. Liu, Y. *et al.* Mutational Mtc6p attenuates autophagy and improves secretory expression of heterologous proteins in Kluyveromyces marxianus. *Microb Cell Fact* 17, 144, doi:10.1186/s12934-018-0993-9 (2018).
2. Hu, Z. *et al.* Discovery and engineering of small SlugCas9 with broad targeting range and high specificity and activity. *Nucleic Acids Res* 49, 4008-4019, doi:10.1093/nar/gkab148 (2021).
3. Longtine, M. S. *et al.* Additional modules for versatile and economical PCR-based gene deletion and modification in Saccharomyces cerevisiae. *Yeast* 14, 953-961, doi:10.1002/(sici)1097-0061(199807)14:10<953::Aid-yea293>3.0.Co;2-u (1998).
4. Shi, T. *et al.* Characterization and modulation of endoplasmic reticulum stress response target genes in Kluyveromyces marxianus to improve secretory expressions of heterologous proteins. *Biotechnology for Biofuels* 14, 236, doi:10.1186/s13068-021-02086-7 (2021).
