# Supplementary material for: Efficient and markerless gene integration with SlugCas9-HF in Kluyveromyces marxianus
Source: Commun Biol. 2024 Jul 2;7:797. doi: 10.1038/s42003-024-06487-w (PMC11219867; doi:10.1038/s42003-024-06487-w)
Supplement: Supplementary file 2 — Description of Additional Supplementary Files [file 42003_2024_6487_MOESM2_ESM.pdf]

Description of Additional Supplementary Files

File name: Supplementary Data 1

Description: The source data behind the graphs in the paper.

File name: Supplementary Data 2

Description: Full sequences of plasmids LHZ1493, LHZ1494 and pRS425-Cas9-2xSapI.
