# Supplementary material for: Efficient and markerless gene integration with SlugCas9-HF in Kluyveromyces marxianus
Source: Commun Biol. 2024 Jul 2;7:797. doi: 10.1038/s42003-024-06487-w (PMC11219867; doi:10.1038/s42003-024-06487-w)
Supplement: Supplementary file 5 — Reporting Summary [file 42003_2024_6487_MOESM5_ESM.pdf]

nature portfolio

Corresponding author(s): Yongming Wang, Yao Yu, Hong Lu

Last updated by author(s): Jun 3, 2024

Reporting Summary

Nature Portfolio wishes to improve the reproducibility of the work that we publish. This form provides structure for consistency and transparency in reporting. For further information on Nature Portfolio policies, see our Editorial Policies and the Editorial Policy Checklist.

Statistics

For all statistical analyses, confirm that the following items are present in the figure legend, table legend, main text, or Methods section.

n/a	Confirmed	
☐	☒	The exact sample size (*n*) for each experimental group/condition, given as a discrete number and unit of measurement
☐	☒	A statement on whether measurements were taken from distinct samples or whether the same sample was measured repeatedly
☐	☒	The statistical test(s) used AND whether they are one- or two-sided<br>*Only common tests should be described solely by name; describe more complex techniques in the Methods section.*
☐	☒	A description of all covariates tested
☐	☒	A description of any assumptions or corrections, such as tests of normality and adjustment for multiple comparisons
☐	☒	A full description of the statistical parameters including central tendency (e.g. means) or other basic estimates (e.g. regression coefficient) AND variation (e.g. standard deviation) or associated estimates of uncertainty (e.g. confidence intervals)
☐	☒	For null hypothesis testing, the test statistic (e.g. *F*, *t*, *r*) with confidence intervals, effect sizes, degrees of freedom and *P* value noted<br>*Give P values as exact values whenever suitable.*
☒	☐	For Bayesian analysis, information on the choice of priors and Markov chain Monte Carlo settings
☒	☐	For hierarchical and complex designs, identification of the appropriate level for tests and full reporting of outcomes
☒	☐	Estimates of effect sizes (e.g. Cohen's *d*, Pearson's *r*), indicating how they were calculated

Our web collection on statistics for biologists contains articles on many of the points above.

Software and code

Policy information about availability of computer code

Data collection	Deep sequencing data were collected using an Novaseq V1.5 software installed in an NovaS4 sequencer(Illumina).
Data analysis	Microsoft Office 365 Excel, and GraphPad Prism 5 were usedfor data averaging, calculation of standard deviations,and evaluation for statistical significance.

For manuscripts utilizing custom algorithms or software that are central to the research but not yet described in published literature, software must be made available to editors and reviewers. We strongly encourage code deposition in a community repository (e.g. GitHub). See the Nature Portfolio guidelines for submitting code & software for further information.

Data

Policy information about availability of data

All manuscripts must include a data availability statement. This statement should provide the following information, where applicable:

- Accession codes, unique identifiers, or web links for publicly available datasets
- A description of any restrictions on data availability
- For clinical datasets or third party data, please ensure that the statement adheres to our policy

The RNA-seq datasets being subjected to analysis in this study can be found in online NCBI Sequence Read Archive (SRA) repositories as follows: https://ncbi.nlm.nih.gov/bioproject/658204. All data generated during the study are available within the article and its Supplementary Information files. Source data are provided in Supplementary Data 1. Uncropped and unedited blot/gel images were provided in Supplementary Information files. Full sequences of plasmids

LHZ1493, LHZ1494 and pRS425-Cas9-2xSapI are provided in Supplementary data 2, and plasmids LHZ1493, LHZ1494 will be available via the non-profit plasmid repository Addgene (Plasmid ID: 222102 and 222104). Any remaining information can be obtained from the corresponding author upon reasonable request

Research involving human participants, their data, or biological material

Policy information about studies with human participants or human data. See also policy information about sex, gender (identity/presentation), and sexual orientation and race, ethnicity and racism.

Reporting on sex and gender	n/a
Reporting on race, ethnicity, or other socially relevant groupings	n/a
Population characteristics	n/a
Recruitment	n/a
Ethics oversight	n/a

Note that full information on the approval of the study protocol must also be provided in the manuscript.

Field-specific reporting

Please select the one below that is the best fit for your research. If you are not sure, read the appropriate sections before making your selection.

☒ Life sciences ☐ Behavioural & social sciences ☐ Ecological, evolutionary & environmental sciences

For a reference copy of the document with all sections, see nature.com/documents/nr-reporting-summary-flat.pdf

Life sciences study design

All studies must disclose on these points even when the disclosure is negative.

Sample size	We determined the sample size based on the specific purpose of the experiment and the number of yeast colonies formed. For indel rates of the 24 sgRNAs detected by deep sequencing, all transformants formed were tested. For integration efficiency at the six selected integration sites, 48 transformants were tested for each of the three independent replicate experiments. For integration efficiency of various lengths(1.5kb, 3kb, 6kb and 12kb) of heterologous genes at the sg1 locus, 48 transformants for Donor- 1,5kb or 32 transformants for Donor-3kb were selected, while for Donor- 6kb and Donor- 12kb, all transformants formed were seclected due to the limited number of colonies formed. For multilocus integration efficiency, 100 transformants were tested. To identify strains with high heme accumulation, 49 transformants were selected for primary screening.
Data exclusions	No data were excluded for analysis.
Replication	Data are presented as mean values and standard deviations of three independent biological replicates. All attempts shown in this study were successful.
Randomization	In our study, yeast colonies from transformations were randomly selected for further analysis.
Blinding	Blinding does not apply to our experiments.

Reporting for specific materials, systems and methods

We require information from authors about some types of materials, experimental systems and methods used in many studies. Here, indicate whether each material, system or method listed is relevant to your study. If you are not sure if a list item applies to your research, read the appropriate section before selecting a response.

Materials & experimental systems

n/a	Involved in the study
☒	☐ Antibodies
☒	☐ Eukaryotic cell lines
☒	☐ Palaeontology and archaeology
☒	☐ Animals and other organisms
☒	☐ Clinical data
☒	☐ Dual use research of concern
☒	☐ Plants

Methods

n/a	Involved in the study
☒	☐ ChIP-seq
☒	☐ Flow cytometry
☒	☐ MRI-based neuroimaging

Plants

Seed stocks	n/a
Novel plant genotypes	n/a
Authentication	n/a
